# Generalized scaling of spin qubit coherence in over 12,000 host materials

**DOI:** 10.1073/pnas.2121808119

**Published:** 2022-04-06

**Authors:** Shun Kanai, F. Joseph Heremans, Hosung Seo, Gary Wolfowicz, Christopher P. Anderson, Sean E. Sullivan, Mykyta Onizhuk, Giulia Galli, David D. Awschalom, Hideo Ohno

**Affiliations:** ^a^Laboratory for Nanoelectronics and Spintronics, Research Institute of Electrical Communication, Tohoku University, Aoba-ku, Sendai 980-8577, Japan;; ^b^Precursory Research for Embryonic Science and Technology (PRESTO), Japan Science and Technology Agency, Kawaguchi 332-0012, Japan;; ^c^Division for the Establishment of Frontier Sciences of Organization for Advanced Studies at Tohoku University, Tohoku University, Aoba-ku, Sendai 980-8577, Japan;; ^d^Center for Science and Innovation in Spintronics, Tohoku University, Aoba-ku, Sendai 980-8577, Japan;; ^e^Center for Spintronics Research Network, Tohoku University, Aoba-ku, Sendai 980-8577, Japan;; ^f^Center for Molecular Engineering and Materials Science Division, Argonne National Laboratory, Lemont, IL 60439;; ^g^Pritzker School of Molecular Engineering, The University of Chicago, Chicago, IL 60637;; ^h^Department of Physics, Ajou University, Suwon, Gyeonggi 16499, Republic of Korea;; ^i^Department of Energy Systems Research, Ajou University, Suwon, Gyeonggi 16499, Republic of Korea;; ^j^Department of Physics, The University of Chicago, Chicago, IL 60637;; ^k^Department of Chemistry, The University of Chicago, Chicago, IL 60637;; ^l^Center for Innovative Integrated Electronic Systems, Tohoku University, Aoba-ku, Sendai 980-0845, Japan;; ^m^World Premier International Research Center Initiative–Advanced Institute for Materials Research, Aoba-ku, Sendai 980-8577, Japan

**Keywords:** quantum information, spin qubits, electron spin coherence, cluster correlation expansion, scaling laws

## Abstract

Spin defect centers with long quantum coherence times (*T*_2_) are key solid-state platforms for a variety of quantum applications. Cluster correlation expansion (CCE) techniques have emerged as a powerful tool to simulate the *T*_2_ of defect electron spins in these solid-state systems with good accuracy. Here, based on CCE, we uncover an algebraic expression for *T*_2_ generalized for host compounds with dilute nuclear spin baths under a magnetic field that enables a quantitative and comprehensive materials exploration with a near instantaneous estimate of the coherence time. We investigated more than 12,000 host compounds at natural isotopic abundance and found that silicon carbide (SiC), a prominent widegap semiconductor for quantum applications, possesses the longest coherence times among widegap nonchalcogenides. In addition, more than 700 chalcogenides are shown to possess a longer *T*_2_ than SiC. We suggest potential host compounds with promisingly long *T*_2_ up to 47 ms and pave the way to explore unprecedented functional materials for quantum applications.

## Significance

Atomic defects in solid-state materials are promising candidates as quantum bits, or qubits. New materials are actively being investigated as hosts for new defect qubits; however, there are no unifying guidelines that can quantitatively predict qubit performance in a new material. One of the most critical property of qubits is their quantum coherence. While cluster correlation expansion (CCE) techniques are useful to simulate the coherence of electron spins in defects, they are computationally expensive to investigate broad classes of stable materials. Using CCE simulations, we reveal a general scaling relation between the electron spin coherence time and the properties of qubit host materials that enables rapid and quantitative exploration of new materials hosting spin defects.

Defect centers have been used to demonstrate a wide range of functionalities (1–5), including remote entanglement (6), control of large nuclear spin clusters (7), and quantum sensing of local temperature (8) and magnetic (9), electric (10), and strain fields (11). While these functionalities have been investigated in only a few solid-state systems, new defects and host materials may offer a new range of opportunities. Weber et al. (1) consolidated the generalized criteria for the preferable properties of materials hosting defect spin qubits (4, 12): a wide bandgap, small spin-orbit coupling, nuclear spin free lattice, and availability of high-quality single crystals. These criteria led to the identification of silicon carbide (SiC) as a promising host for qubits (12–21), which broadened the field beyond the negatively charged nitrogen-vacancy (NV^−^) center in diamond and uncovered a varied landscape of materials for defect spin qubits with different relative advantages and disadvantages (15, 20).

For most quantum applications, the key property of interest is the electron spin coherence time, generally defined as $T_{2}$ by Hahn echo measurement (i.e., after refocusing of slow fluctuating noise by a single π-pulse) (14). Generally, the electron spin $T_{2}$ is limited by the interaction of the spin with its surrounding electric, thermal, and magnetic noise. However, in the absence of additional paramagnetic defects or spin relaxation time ($T_{1}$) limitations, in most quantum applications, the electron spin $T_{2}$ is well predicted by considering only the effect of nuclear spins in the host materials, especially in high-quality, wide-bandgap crystals at cryogenic temperatures. For an S = 1/2 electron spin interacting with a few I = 1/2 nuclear spins, analytical solutions for the electron spin echo envelope modulation have existed for half a century (22). Unfortunately, a quantitative expression is absent for efficiently predicting $T_{2}$ of a typical electron spin in a solid-state defect center interacting with several thousand nuclear spins (16, 23–26), which is highly desirable in the wide-range search of new quantum host materials.

**Fig. 1.**
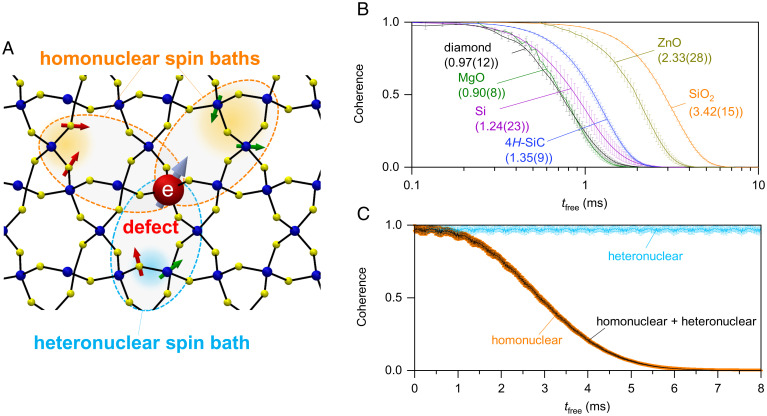
Quantum spin coherence simulation. (*A*) Schematic of CCE-2 of a defect electron spin in a heteronuclear compound. Arrows indicate nuclear (red and green) and electron (skyblue) spins with finite quantum numbers. (*B*) Hahn echo signal $L(t_{\text{free}})$ versus free evolution time $t_{\text{free}}$ calculated by CCE-2 for naturally abundant isotopic diamond, 4*H*-SiC, silicon, and several oxides obtained by simulation under external magnetic field B = 5 T. (*C*) $L(t_{\text{free}})$ of SiO_2_ (*α*-quartz) with B = 300 mT. In addition to the $L(t_{\text{free}})$ with dipole–dipole interactions with all baths (black), that with solely homonuclear spin bath (orange) and heteronuclear spins (blue) are shown. Error bars indicate the sample SD of the Hahn echo signal for different instances of nuclear spin coordinates.

Cluster correlation expansion (CCE) (16, 23–25, 27–29) enables accurate calculations of the $T_{2}$ of an electron spin interacting with a large number of nuclear spins by dividing the spins into small subsets of interacting spin clusters (see Fig. 1*A*). In particular, the pairwise treatment of nuclear spins has been shown to provide excellent accuracy in simulating the decoherence of spin qubits in several dilute nuclear spin host materials: Bi dopants in silicon (28), the NV^−^ center in diamond (25), and the neutral divacancy (VV^0^) center in SiC (16). CCE calculations, however, are still not an easy-to-use prediction scheme, requiring derivations from first principles calculations (30, 31) and computationally expensive simulations especially for compounds with I > 1/2, limiting their use for high-throughput searches of new qubit host materials.

Here, we use CCE to uncover a method not only to explore over 12,000 host materials for quantum applications and to discover candidates with a long electron spin coherence time but more importantly to also expand viable quantum materials options by providing an easy-to-use $T_{2}$ prediction scheme. We first investigate how materials with a dilute (<10^22^ cm^−3^) nuclear spin bath comprising one or multiple nuclear spin species can be decomposed into separate independent baths for each species. We then show that the electron spin $T_{2}$ of each individual bath is scaled by its nuclear spin *g*-factor value, density, and quantum number regardless of the crystalline structure of the material. This results in a single phenomenological expression for estimating any compound’s $T_{2}$ without treating the spin Hamiltonian and the time evolution of spins exactly. By calculating $T_{2}$ for every element in the periodic table and mining materials databases (32, 33), we categorize, calculate, and predict many candidates with long quantum coherence times. Even though $T_{2}$ can be limited by interactions other than those with the nuclear bath, our results set the fundamental materials limit for spin decoherence when all other sources are eliminated, in the absence of dynamical decoupling and isotopic purification.

## Results and Discussion

To begin, we benchmarked our CCE calculations (*Materials and Methods*) on known materials. Fig. 1*B* shows the examples of calculated Hahn echo signal ($L(t_{\text{free}})$) using CCE as a function of the free evolution duration ($t_{\text{free}}$) in naturally abundant 4*H*-SiC, diamond, and Si, as well as typical wide bandgap oxides with B = 5 T. We neglect the Fermi contact terms of the short-range hyperfine interaction given the localized electronic nature of deep-level defects and a dilute nuclear spin density in the host. This assumption is supported by the close match with previous CCE calculations on diamond and SiC that reproduce the experimentally obtained coherence times (16, 25). We ignore the quadrupole interaction, whose main effect increases the central spin’s $T_{2}$ by up to several tens of percent. As such, our calculations without quadrupolar terms represent a lower bound on $T_{2}$. The quantitative evaluation of quadrupole interaction is discussed in *SI Appendix*, section 5. We also adopt the secular approximation for the hyperfine interaction, which holds when $S_{z}$ is a good quantum number in the presence of a strong B. Within this approximation, the Hamiltonian is reduced into bath Hamiltonians treating only the nuclear spin bath (16), meaning the calculation is mostly agnostic to the spin defect Hamiltonian. This is crucial to allow for wide-scale predictions.

$T_{2}$ is obtained by fitting the calculated $L(t_{\text{free}})$ with a decay function $e^{-{\left(t_{\text{free}}/T_{2}\right)}^{\eta }}$, where η is a stretching exponent (34). The envelope of the Hahn echo signal is critically determined by the dipole–dipole interactions between nuclear spins. Fig. 1*C* shows the $L(t_{\text{free}})$ of SiO_2_ (*α*-quartz) with B = 300 mT, dividing the interactions between baths of homo- and heteronuclear spins in the simulation. Heteronuclear spin interactions do not contribute to decoherence in this time range, supporting that the homonuclear spin–spin interaction is the main source of decoherence due to the decoupling of the heteronuclear spin baths. Generally, when the heteronuclear dipole–dipole interactions are much smaller than the difference of their Zeeman energies, the heteronuclear spin baths are decoupled (26). We find that even in a worst-case scenario, any heteronuclear spin baths can be decoupled under reasonable experimental conditions (*Materials and Methods*).

When heteronuclear spin baths are decoupled, one can simulate a compound’s Hahn echo signal by considering only the homonuclear spin baths; $L(t_{\text{free}})$ is calculated by $\prod_{i}^{\;}L_{i}(t_{\text{free}})$ simulating the Hahn echo signal ($L_{i}(t_{\text{free}})$) of isotope i, resulting in approximated $T_{2}$ of a compound by that of isotope i ($T_{2,i}$)

[1] $$T_{2}\approx {\left(\underset{i}{\sum }T_{2,i}{}^{-\eta_{i}}\right)}^{-\frac{1}{\eta^{\prime }}},$$

with $\eta_{i}$ and $\eta^{\prime }$ assumed to be 2 in most cases (*Materials and Methods*).

**Fig. 2.**
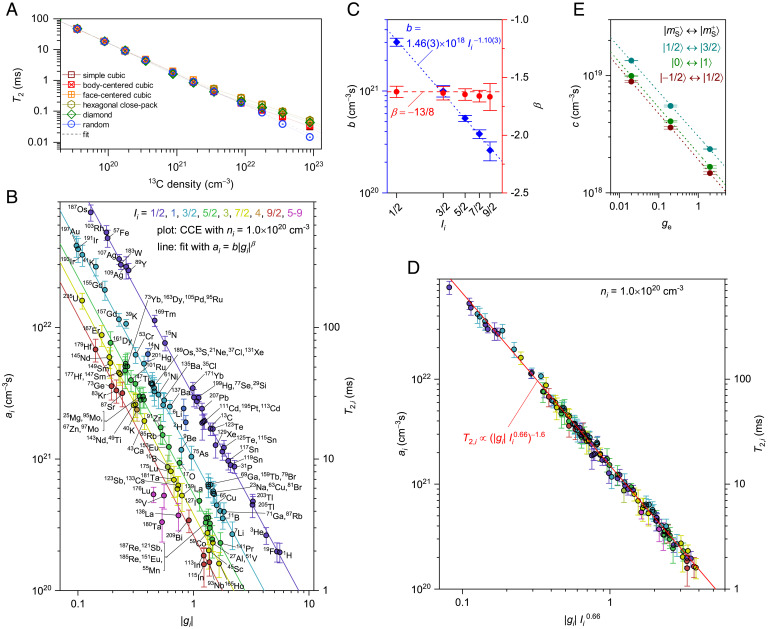
Scaling of quantum coherence of decoupled spin baths. (*A*) Predicted quantum coherence time $T_{2}$ of defects in crystals composed of carbon as a function of ^13^C density $n_{i}$(i = ^13^C) with various crystal structures. The dashed line shows the fit to a power law $a_{i}n_{i}^{\alpha }$, with $a_{i}$ being coefficient, α the exponent −1.0. An external magnetic field of 5 T is applied along the [111] direction of the diamond structure and along [001] directions of other crystal structures. (*B*) Coefficient $a_{i}$ and corresponding $T_{2}$ with nuclear spin density $n_{i}$ = 1.0×10^20^ cm^−3^ as a function of the absolute value of nuclear spin *g*-factor $|g_{i}|$ calculated for all stable isotopes with the nuclear spin quantum number $I_{i}$. Lines are power law fits $T_{2,i}=b{|g_{i}|}^{\beta }$ on the different half-integer–$I_{i}$ spins. (*C*) Intercept b versus $I_{i}$ with the power law fit $b=cI_{i}^{-1.10\pm 0.03}$ (blue), with c being the coefficient, and the exponent β versus $I_{i}$ with the theoretical value β = −13/8 for $I_{i}$ = 1/2 (25, 27)) (red). (*D*) $T_{2}$ versus $|g_{i}|I_{i}{}^{0.66}$. The solid line is the power law fit. All simulations are conducted under external magnetic field of 5 T. (*A–D*) Electron *g*-factor $g_{\text{e}}$ = 2.0 and S = 1/2 are assumed. (*E*) Coefficient c for the transition of electron spin states between $|m_{\text{S}}^{-}\rangle ↔|m_{\text{S}}^{+}\rangle$ as a function of $g_{\text{e}}$. Dashed lines are the power law fits. Error bars indicate the sample SD obtained by the simulation for different crystal coordinates for the isotopes (*B*, *D*). Error bars indicate the SE obtained from fitting the simulated CCE data (*C*, *E*).

The electron spin $T_{2,i}$ depends mainly on the spin density ($n_{i}$) of nucleus i, the crystalline structure, the nuclear spin *g*-factor ($g_{i}$), and the nuclear spin quantum number ($I_{i}$). We computed $T_{2,i}$ with different $n_{i}$, crystalline structure, and B. The nuclear spin density and crystalline structure dependences of $T_{2,i}$ for ^13^C are shown in Fig. 2*A*. For $n_{{\;}^{13}\text{C}}$ < 10^22^ cm^−3^ (*cf*. natural abundance in diamond: 1.9 × 10^21^ cm^−3^), $T_{2{,}^{13}\text{C}}$ is well fitted by the power law $a_{{\;}^{13}\text{C}}\;n_{{\;}^{13}\text{C}}{}^{-1.0}$, where $a_{{\;}^{13}\text{C}}$ is the coefficient of the power law. The scaling exponent −1.0 reproduces previous CCE simulations for diamond and SiC (16, 35). Most importantly, at this density $T_{2{,}^{13}\text{C}}$ is *independent* of the crystal structure and is governed by interactions between “randomly” positioned ^13^C nuclei. As $n_{\text{spin}}$ increases above 10^22^ cm^−3^, the effect of anisotropy of the dipole–dipole interaction (25, 28, 36) becomes relevant and $T_{2}$ deviates from the power law except in the amorphous limit.

As an estimate in the dilute limit, we can therefore scale $T_{2,i}$ as $a_{i}\;n_{i}{}^{-1.0}$, where the coefficient $a_{i}$, dependent on $g_{i}$ and $I_{i}$, is derived by a fit with the power law to the calculated $T_{2,i}$ versus $n_{i}$ as shown in Fig. 2*A*. Fig. 2*B* then presents $a_{i}$ for all stable isotopes computed by CCE and the corresponding $T_{2,i}$ at $n_{i}$ = 1.0 × 10^20^ cm^−3^ as a function of $g_{i}$. The calculated data line up well with different series of $I_{i}$. The lines are the power law fits $a_{i}=b{|g_{i}|}^{\beta }$, with the coefficient b and the exponent β. Fig. 2*C* summarizes b versus $I_{i}$ and the exponent β versus $I_{i}$. For spin-1/2 isotopes, β has been analytically calculated to be −13/8 ∼ −1.63 (7), which is shown as a dashed line in Fig. 2*C*, and is in good agreement with numerically obtained β = −1.64 ± 0.07 within the error bar regardless of the $I_{i}$. We found that b changes with $I_{i}^{\;}$ and is fitted by the power law $b\propto I_{i}^{-1.10\pm 0.03}$ as shown by the dotted line, which indicates that $T_{2,i}$ can be expressed by ${\left(|g_{i}^{\;}|I_{i}^{\;}{}^{0.66}\right)}^{-1.6}$. Fig. 2*D* shows $T_{2,i}$ versus $|g_{i}^{\;}|I_{i}^{\;}{}^{0.66}$, where all the isotopes of all the elements collapse into one line within the error bars. From fitting with a power law, we determined the phenomenological expression of $a_{i}$ for all isotopes as $a_{i}=c{|g_{i}^{\;}|}^{-1.6}I_{i}^{\;}{}^{-1.1}$, with c being an isotope *independent* constant = 1.5 × 10^18^ cm^−3^s. We therefore obtained the simple expression for $T_{2,i}$ with scaling factors $g_{i}^{\;}$, $I_{i}^{\;}$, and $n_{i}$ (in cm^−3^) as

[2] $$T_{2,i}=1.5\times 10^{18}\;{|g_{i}^{\;}|}^{-1.6}\;I_{i}^{\;}{}^{-1.1}\;n_{i}{}^{-1.0}\;(\text{s}).$$

This expression, obtained by considering CCE of all stable isotopes, combined with Eq. 1 enables an instantaneous estimate of the defect’s $T_{2}$ within any host material without treating defect or bath Hamiltonians exactly, valid for dilute nuclear spin baths. This results in a comprehensive prediction of materials with long $T_{2}$ without the need for any CCE simulations, even for high I, or in complex heteronuclear systems. This quantitatively derived scaling relation indicates that not only $n_{i}$, but also more importantly $g_{i}^{\;}$ and $I_{i}^{\;}$, have a critical effect on the coherence time.

We have assumed defect centers with electron *g*-factor $g_{\text{e}}=2$ and S = 1/2 above, while for S > 1/2 centers, a two-level system (qubit) can be assigned to a given electron spin transition, acting similarly but not equivalently to S = 1/2 under the secular approximation (*Materials and Methods*). For S = 1, for example, $T_{2}$ is shown to be ∼10% longer than that for S = 1/2 through CCE calculations (16) (*SI Appendix*, section 10). Using a generalized fictitious spin for the magnetic dipole transition $|m_{\text{S}}^{-}\rangle ↔|m_{\text{S}}^{+}\rangle$ and recalculating using CCE, we found an expansion of Eq. 2 that modifies its constant prefactor c with different $g_{\text{e}}^{\;}$ for S = 1/2 to 3/2 transitions as shown in Fig. 2*E*. Dashed lines are the fits to power laws $c\propto g_{\text{e}}^{\delta }$, and the exponent δ is ∼ −0.39, which is in good agreement with theoretically obtained value for S = 1/2 and I = 1/2 as −3/8 ∼ −0.38 (25). Note that although $g_{\text{e}}$ can be anisotropic depending on the symmetry of the crystal structure and/or the presence of strong spin-orbit interaction, the scaling holds for the anisotropic $g_{\text{e}}$ under the secular approximation (see *Materials and Methods*). Likewise, $T_{2}$ can also be anisotropic and can depend on the direction of the external magnetic field. This is therefore a universal coherence time holding for all transitions for electron spin centers with a dilute spinful nuclear host (*SI Appendix*, sections 8 and 9). This expression also hints toward further possible theoretical work that may unravel the physics behind this universal scaling.

**Fig. 3.**
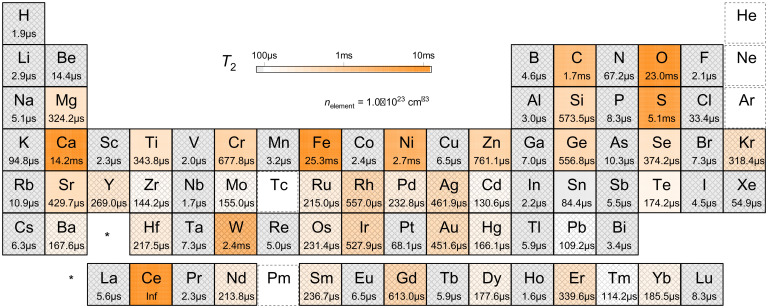
Periodic table for quantum coherence. Coherence time $T_{2}$ based on CCE calculations for spin qubits in hypothetical material hosts with natural abundance of a single species with element density $n_{\text{element}}$ = 1.0×10^23^ cm^−3^ obtained by Eqs. 1 and 2 at the dilute limit assuming an electron spin *g*-factor of 2 and quantum number of 1/2. Hatched elements contain spinful nuclear spin density over the dilute limit $n_{\text{i}}$ ∼1.0×10^22^ cm^−3^ at $n_{\text{element}}$ = 1.0×10^23^ cm^−3^. Note that diamond has one of the largest number densities in compounds with $n_{\text{C}}$ = 1.8×10^23^ cm^−3^, and $n_{\text{element}}$ of each element in compound is smaller than 1.0×10^23^ cm^−3^. The periodic table is color coded by $T_{2}$ on a log scale. Materials that are difficult to make compounds from (He, Ne, Ar) or that are without stable isotopes (Tc, Pm) are excluded.

In order to prepare for a wide-scale exploration of coherence times for host materials, we investigated the $T_{2}$ of every element in the periodic table, assuming a natural abundance of isotopes, as shown in Fig. 3, taking the element density ($n_{\text{element}}$) of 1.0 × 10^23^ cm^−3^ based on the scaling relationship in Eqs. 1 and 2 and assuming an electron spin *g*-factor of 2. This table provides a unique lens to explore the materials engineering guidelines for synthesizing quantum-relevant materials with tailored spin coherence properties. Among the elements that form solid compounds, only cerium has no effect on $T_{2}$ because all stable isotopes have $I_{i}$ = 0. In addition, there are seven elements with longer coherence times than carbon, which suggests their allotropes or compounds could yield longer coherence time than that of diamond spin centers.

Finally, we demonstrated a comprehensive prediction of $T_{2}$ based on Eqs. 1 and 2. We utilized structural information from online databases (32, 33) to automate the process, considering 12,847 stable materials with first principles–predicted bandgaps larger than 1.0 eV (Datasets). Table 1 shows the list of the materials with $T_{2}$ > 10 ms. Here, we assumed materials have natural isotopic abundance. In addition to $T_{2}$ and bandgap, there are naturally other material considerations to be made when exploring new host materials. The list is screened in its generality so as to not impose too many material restrictions to minimize any bias to the study of the materials. Thus, for example, we have not screened materials based on their magnetism, Debye temperature, and dimensionality. We attribute the slight deviations of the values on Table 1 from a full CCE calculation in Fig. 1*B* to the error on the exponents in Eq. 2, the anisotropy of the dipole–dipole interaction, and the fact that η is approximated to be 2, as discussed in *Materials and Methods* and *SI Appendix*, section 7. However, the calculated difference is ∼20% and does not hinder screening materials for quantum coherence. We found that CeO_2_ has the largest $T_{2}$ of all investigated materials at 47 ms, which is virtually the upper limit of $T_{2}$ for all naturally abundant compounds. Beyond choosing the elements of the host crystal and reducing the dimensionality of the host (35), isotopic purification of the material (39–41) can further extend $T_{2}$ coherence times; however, isotopic purification of certain materials is often cost prohibitive or impossible depending on isotopic species. While dynamical decoupling can also increase coherence, inherent limitations from control fidelities highlight the importance of starting with a long coherence time before applying these techniques. Independent of the host material, some spin defect systems are inherently associated with a nuclear spin, and the local hyperfine interaction would mix the electron and nuclear spins, which could be beneficial to prolonging the spin coherence time (14). However, for some cases (e.g., in dynamical decoupling, clock transitions, or low dimensional systems), the extent to which magnetic noise-limited coherence may be extended has a strong correlation with the Hahn echo coherence time of the bare electron spin in a three-dimensional system, as studied here.

**Table 1.** Top quantum coherence time *T*_2_ materials obtained by **Eqs. 1** and **2** at the dilute limit assuming an electron spin *g*-factor of 2 and quantum number of 1/2

No.	Material	*T*_2_ (ms)	Crystal system	Θ_Debye_ (K)	Notes
1	CeO_2_	47	Cubic	448	
2	FeO	36	Monoclinic	298 (37)	Antiferromagnetic
3	CaO	34	Cubic	646	
4	CaSO_4_	29	Orthorhombic	—	
5	Ce(SO_4_)_2_	29	Orthorhombic	—	
6	SO_3_	29	Orthorhombic	—	*K*_Reuss_ ∼5 GPa
7	FeSO_4_	28	Orthorhombic	—	Ferromagnetic
8	CaS_3_O_10_	28	Monoclinic	—	
9	Ca_3_WO_6_	27	Trigonal	—	
10	WS_2_O_9_	25	Monoclinic	—	
11	Ca_2_FeWO_6_	24	Monoclinic	—	FerromagneticnW 183 = 1.2 × 10^21^ cm^−3^
12	CaS	23	Cubic	449	
13	Ca_2_NiWO_6_	19	Monoclinic	—	AntiferromagneticnW 183 = 1.2 × 10^21^ cm^−3^
14	S	19	Monoclinic	—	
15	CaWO_4_	18	Tetragonal	335 (38)	nW 183 = 1.8 × 10^21^ cm^−3^
16	CS_14_	18	Trigonal	—	
17	Fe_2_NiO_4_	18	Orthorhombic	—	Ferromagnetic
18	S_8_O	17	Orthorhombic	—	*K*_Reuss_ ∼1 GPa
19	FeWO_4_	16	Monoclinic	405	FerromagneticnW 183 = 2.0 × 10^21^ cm^−3^
20	NiSO_4_	15	Orthorhombic	—	Antiferromagnetic
21	WO_3_	13	Tetragonal	529	nW 183 = 2.5 × 10^21^ cm^−3^
22	NiWO_4_	12	Monoclinic	—	AntiferromagneticnW 183 = 2.1 × 10^21^ cm^−3^
23	WS_2_	11	Trigonal	—	2D material*K*_Reuss_ ∼2 GPanW 183 = 2.3 × 10^21^ cm^−3^
24	Sr_2_Si(S_2_O_7_)_4_	11	Monoclinic	—	
25	Sr_2_Ge(S_2_O_7_)_4_	11	Monoclinic	—	
26	CaCO_3_	11	Trigonal	502	
					
138	SiO_2_	2.7	Tetragonal	523	
298	ZnO	1.9	Hexagonal	398	
709	SiC (4*H*)	1.1	Hexagonal	1147	
936	C (diamond)	0.89	Cubic	2217	nC 13 = 1.9 × 10^21^ cm^−3^
1,125	MgO	0.60	Cubic	900	nM 29g = 5.2 × 10^21^ cm^−3^

Materials with *T*_2_ > 10 ms and bandgap > 1 eV, as well as those listed in Fig. 1*B*, are shown. Crystal system, Debye temperature Θ_Debye_, and other specific material properties (e.g., magnetism [ferromagnet/antiferromagnet], hardness [soft materials with predicted bulk modulus (Reuss average) *K*_Reuss_ < 10 GPa], dimensionality, and spinful nuclei density *n*_i_ [relatively high *n*_i_ > 10^21^ cm^−3^] are noted for the practical use.) See datasets (45, 46) for details.

**Fig. 4.**
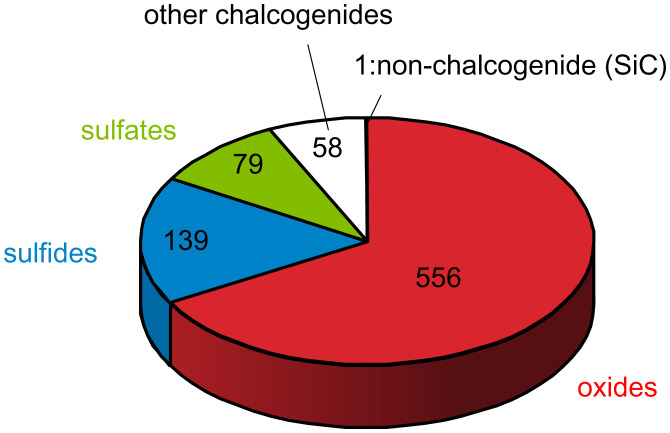
Materials to explore. Types of 832 stable compounds with quantum coherence time $T_{2}$ longer than 1 ms and predicted bandgap larger than 1.0 eV. SiC is the only stable widegap nonchalcogenide with $T_{2}$ > 1 ms.

Of the compounds considered, there are 27 materials with natural isotopic abundance with coherence times longer than 10 ms, all of which are composed of oxides, sulfides, and sulfates. Fig. 4 shows the types of all 832 materials with $T_{2}$ > 1 ms. SiC has the longest $T_{2}$ among nonchalcogenides, and our results point to the exploration of chalcogenide materials for longer $T_{2}$ times than SiC.

## Conclusion

We offer a simple, high-throughput method to predict coherence times for spin defects to screen possible quantum host materials. This is achieved by uncovering a general scaling behavior for any S, $I_{i}$, $g_{\text{e}}^{\;}$, $g_{i}^{\;}$, and $n_{i}^{\;}$ in the dilute limit for spin coherence in solids that depends on the effective coherence times of a compound’s constituent isotopes. The scaling relation here can be applied to the isotopically purified materials as well, providing a predictive tool guiding materials growth and purity requirements. While we do not fully account for geometric factors, such as in two-dimensional (2D) materials (35), we have demonstrated that in the absence of magnetic ordering, the coherence time for bulk materials depends only on the nuclear spin *g*-value, its spin quantum number, and density, regardless of crystalline structure for these dilute nuclear spin compounds. The predictive power of this expression points to 27 materials with coherence times longer than 10 ms and to oxides or sulfides with Ce, Fe, Ca, and Ni as cations as promising long coherence time hosts from the standpoint of nuclear spins. In combination with data mining approaches (42), these results present potential materials systems with promisingly long coherence times and pave the way to explore unprecedented and varied functional materials for quantum applications.

## Materials and Methods

### Spin Hamiltonian, Density Matrix, and Its Time Evolution.

We considered the spin Hamiltonian H defined by

[3] $$H=H_{\text{S}}+H_{\text{B}}+H_{\text{S}-\text{B}},$$

where $H_{\text{S}}$ and $H_{\text{B}}$ are Hamiltonians for electron spin and nuclear spins, respectively, and $H_{\text{S}-\text{B}}$ indicates electron spin–nuclear spin interaction (16, 36, 43).

[4] $$H_{\text{S}}=-g_{\text{e}}\mu_{\text{B}}BS_{z},$$

[5] $$H_{\text{B}}=-\underset{i}{\sum }g_{i}\mu_{\text{N}}BI_{z,i}+H_{\text{n}-\text{n}},$$

[6] HS−B≈μ04πgeμBμNS→⋅∑igi[I→iri3−3(I→i⋅ri→)ri→ri5] ≈Sz∑iA→i⋅I→i,

where $g_{\text{e}}$, $g_{i}$, $\mu_{\text{B}}$, $\mu_{\text{N}}$, and $\mu_{0}$ are the *g*-factor of the electron, the *g*-factor of nuclear spin of nucleus i, Bohr magneton, nuclear magneton, and the permeability of vacuum, respectively. We set the magnetic field direction along the z direction and the electron spin quantum number to be 1/2. $\vec{r_{i}}$, $r_{i}$, B, ${\vec{A}}_{i}$, $\vec{S}$, $S_{z}$, ${\vec{I}}_{i}$, and $I_{z,i}$ are the vector from electron spin to the nucleus i, $|\vec{r_{i}}|$, the magnetic field, hyperfine field vector of nucleus i, the electron spin vector operator, z component of electron spin operator, the spin vector operator of nucleus i, and z component of the spin operator vector of nucleus i, respectively. $H_{\text{n}-\text{n}}$ is the Hamiltonian of nuclear spin–nuclear spin interactions:

[7] $$H_{\text{n}-\text{n}}=\frac{\mu_{0}}{4\text{π}}\mu_{\text{N}}^{2}\underset{i,j}{\sum }g_{i}g_{j}\left[\frac{{\vec{I}}_{i}\cdot {\vec{I}}_{j}}{r_{ij}^{3}}-\frac{3\left({\vec{I}}_{i}\cdot {\vec{r}}_{ij}\right)\left({\vec{I}}_{j}\cdot {\vec{r}}_{ij}\right)}{r_{ij}^{5}}\right],$$

where ${\vec{r}}_{ij}$ is the vector from nucleus i to nucleus j and $r_{ij}=|{\vec{r}}_{ij}|$. Two of the approximations in Eq. **6** are valid when 1) the Fermi contact term is negligible with a localized electron spin center and dilute nuclear spins in the host, which is valid in most of the intrinsic and extrinsic defects in, for example, SiC and diamond and 2) two of the electron spin states $m_{\text{S}}$ = ±1/2 are of order GHz (e.g., when one applies, for the $g_{\text{e}}=2$ defects, a magnetic field larger than 30 mT, which is a standard measurement condition for the pseudospin model). Among all the simple substances, diamond has the largest number density (1.8 × 10^23^ cm^−3^), and the effect of the Fermi contact terms on the spin coherence time is larger than the dipole–dipole interaction only when ^13^C is enriched to over 10% (40) (1.8 × 10^22^ cm^−3^). Furthermore, the materials list we show is mainly composed of oxides, sulfides, and sulfates with natural nuclear spin abundance, in which the nuclear spin number density is much smaller than ^13^C in the diamond enriched at 10% abundance. Therefore, for deep defects like the NV in diamond, the Fermi contact term is negligible in our calculations of dilute nuclear spin compounds.

We note that depending on the symmetry of the crystal structure and/or the presence of strong spin-orbit interaction, $g_{\text{e}}$ can be anisotropic. In this case, the coherence time can be modulated by the direction of the external magnetic field. Under the secular approximation, Eqs. **4****–****7** hold for the anisotropic *g*-factor of the electron spin.

Under the secular approximation, the electron spin operator with S>1/2 can be treated as a pseudospin. When we consider a generic coherence $|m_{\text{S}}\rangle :\;|m_{0}-1/2\rangle ↔|m_{0}+1/2\rangle$ ($m_{0}$: half-integer), $S_{z}$ is defined as a 2×2 matrix with components ${\text{δ}}_{l,m}(m_{0}\mp 1/2)$, where ${\text{δ}}_{l,m}$ is Kronecker’s delta. For example, for $m_{0}=1/2\;(|m_{\text{S}}\rangle :|0\rangle ↔|1\rangle )$, which represents a spin with an integer electron spin quantum number, we utilized a partial matrix of the spin operator ${S^{\prime }}_{z}=\left[\begin{array}{cc} 0 & 0\\ 0 & 1 \end{array}\right]$, which gives an offset $\left[\begin{array}{cc} 1/2 & 0\\ 0 & 1/2 \end{array}\right]$ in $S_{z}$ and the hyperfine coupling S⋅I, resulting in the bias fields to the nuclear spins.

Time evolution of the density matrix $\rho (t_{\text{free}})$ is calculated by

[8] $$\rho \left(t_{\text{free}}\right)=U\left(t_{\text{free}}\right)\rho \left(0\right)U^{†}\left(t_{\text{free}}\right).$$

We used the standard Hahn echo propagator composed of ${\left(\pi /2\right)}_{x}$ pulse, free evolution for $t_{\text{free}}/2$, ${\text{π}}_{x}$ pulse, and free evolution for $t_{\text{free}}/2$ as

[9] $$U\left(t\right)=\exp\;\left(-i\frac{H}{\hbar }\frac{t_{\text{free}}}{2}\right)\exp\;\left(i\text{π}S_{x}\right)\exp\;\left(-i\frac{H}{\hbar }\frac{t_{\text{free}}}{2}\right)\exp\;\left(i\frac{\text{π}}{2}S_{x}\right).$$

The initial density matrix is taken to be $\rho \left(0\right)=\rho_{\text{S}}\left(0\right)⊗\rho_{\text{B}}\left(0\right)$ using electron spin projected density matrix $\rho_{\text{S}}\left(0\right)$ with z projection of the spin $m_{\text{S}}=-1/2$ state

[10] $$\rho_{\text{S}}\left(0\right)=|-\frac{1}{2}\rangle \langle -\frac{1}{2}|,$$

and bath projected density matrix $\rho_{\text{B}}\left(0\right)$

[11] $$\rho_{\text{B}}\left(0\right)=\sum_{I}P_{I}|I\rangle \langle I|,$$

with $P_{I}$ being the probability of the nuclear state ∣I〉. Hahn echo signal $L\left(t_{\text{free}}\right)$ is calculated by

[12] $$L\left(t\right)=\frac{\text{Tr}\left[\rho \left(t_{\text{free}}\right)S_{+}\right]}{\text{Tr}\left[\rho \left(0\right)S_{+}\right]},$$

where $S_{+}$ is raising operator of electron spin (22).

### CCE Calculation.

Hahn echo signal $L^{\text{CCE}-1}$ obtained by first- and second-order CCE (CCE-1 and CCE-2, respectively) calculations are defined as (23)

[13] $$L^{\text{CCE}-1}=\prod_{i}L_{i},$$

[14] $$L^{\text{CCE}-2}=L^{\text{CCE}-1}\prod_{i,j}\frac{L_{i,j}}{L_{i}L_{j}},$$

where $L_{i}$ ($L_{i,j}$) is the Hahn echo signals calculated with the central electron spin and the *i*-th nuclear spin (electron spin and the *i*-th and *j*-th nuclear spins). We have confirmed that in the dilute nuclear spin bath like the compounds in Table 1, the effect of the three or higher body spin interaction is negligible, and the $L(t_{\text{free}})$ converges with CCE-2 (*SI Appendix*, section 3) as with the previous report on the naturally isotopic diamond and 4*H*-SiC (16, 35). In Fig. 1 *B* and *C* and *SI Appendix*, Fig. S2, Hahn echo signals are calculated for 5 to 10 different sets of nuclear spin coordinates randomly placed on lattice sites with the natural nuclear abundance, and their average and SD of the echo signals are shown by the symbol and the error bar, respectively. Fig. 2 *B* and *D* and *SI Appendix*, Fig. S6 show calculated *T*_2_ of hypothetical host materials composed of a single element with one of the stable crystal structures (Fe, W: bcc/Au, Cu: fcc/Be, Co: hcp/C, Si: diamond/etc.) and their reported lattice constants. There, each nuclear spin abundance is taken to realize the nuclear spin density *n*_i_ = 1 × 10^20^ cm^−3^ considering their crystal structures and lattice constants. The average and SD of *T*_2_ with 10 different random nuclear spin coordinations on the lattice site are shown by the symbol and error bar, respectively.

### Decoupling Field.

The envelope of the Hahn echo signal is critically affected by the dipole–dipole interactions between nuclear spins. The dipole–dipole interaction between heteronuclear spins is characterized by two factors: Ω and δ. Ω indicates the dipole–dipole interactions between nucleus i and j, which is given by Eq. 7. δ indicates the energy splitting between two levels interacting with $I_{+,i}I_{-,j}+I_{-,i}I_{+,j}$ due to the different Zeeman splitting with different nuclear spin *g*-factors between nuclei in addition to the dipole–dipole interaction between them, with $I_{\pm ,i}$ being the ladder operator of spin in nucleus i given by Eq. 5. When δ≫Ω, the heteronuclear spin baths are decoupled. Considering $I_{+,i}I_{-,j}+I_{-,i}I_{+,j}$ is the main source of the decoherence (16), we estimated decoupling field $B_{\text{dec}}$ as

[15] $$B_{\text{dec}}=\frac{\mu_{0}}{4\text{π}}\mu_{\text{N}}\frac{1}{l^{3}}\frac{g_{i}g_{j}}{g_{i}-g_{j}},$$

with l being the distance of the nearest-neighbor nucleus i and nucleus j (*SI Appendix*). For example, $B_{\text{dec}}$ is 0.28 mT (0.13 mT) for SiO_2_ (SiC), above which the heteronuclear spin baths decouple (34, 44).

Using CCE calculations, Seo et al. (16) have numerically shown that B < 30 mT decouples heteronuclear spin baths assuming the difference of nuclear spin *g*-factor values (Δg) = 0.021 and l = 1.3 Å (8). These Δg and l values are relatively small among the compounds. Also in experiments, B up to 300 mT ∼1 T is achievable with a standard yoke magnet. In Eq. 15, the decoupling field $B_{\text{dec}}$ is proportional to $1/l^{3}\text{Δ}g$, thus suggesting the heteronuclear spin baths are decoupled in most of the compounds under standard experimental conditions.

As example systems, let us consider the oxide and sulfides. The ionic radius of the O^2−^ is 0.14 nm at minimum; thus, $B_{\text{dec}}$ is estimated to be ∼$g_{\text{O}}^{2}/\text{Δ}g$ × 0.9 mT at most by Eq. 15, with Δg being the difference of the *g*-factors between ^17^O and cation. For the worst case among all isotopes, Δg = 0.024 for ^9^Be gives a maximum $B_{\text{dec}}$ ∼5 mT. For sulfides, the largest $B_{\text{dec}}$ is given by ^189^Os with Δg = 0.011, as ∼3 mT. Note that the magnetic field > $B_{\text{dec}}$ used in the exploration of the material is typically larger than the magnetic field to operate clock transitions, where d*f*/d*B* (*f*: resonance frequency) is mainly determined by the electron spin *g*-factor (14, 28).

### Stretching Exponent.

A compound’s $T_{2}$ is defined by each isotope’s coherence time ($T_{2,i}$) by the condition $\sum_{i}{\left(T_{2}/T_{2,i}\right)}^{-\eta_{i}}=1$, where $\eta_{i}$ is the stretching exponent for the $L_{i}(t)$. We found this $T_{2}$ is well approximated by

[16] $$T_{2}\approx {\left(\underset{i}{\sum }T_{2,i}{}^{-\eta_{i}}\right)}^{-\frac{1}{\eta^{\prime }}},$$

with $\eta_{i}$ and $\eta^{\prime }$ assumed to be 2. For example, when $T_{2,j}=T_{2,i}/10$ ($T_{2,j}=T_{2,i}/3$), $T_{2}$ in binary compounds with nucleus i and j obtained by Eq. 1 with $\eta_{i}$ = $\eta^{\prime }$ = 2 deviates from the exact $T_{2}$ by 0.44% (4.0%) at the very most among the typical $\eta_{i}$ and $\eta_{j}$ values 2 to 3 (8, 16).

### Materials Explorations.

For $T_{2}$ prediction, we used crystallographic information framework (CIF) files available at The Materials Project (32, 33). From CIF files, $n_{i}$ is derived and $T_{2}$ is calculated by using Eqs. 1 and 2. Only the predicted but realistic and stable materials (i.e., materials with zero-energy above hull) are calculated. Most of the crystallographic data are obtained by calculation at 0 K or are based on the experimental result measured at room temperature. The thermal expansion coefficient is in the order of 10^−6^ to 10^−5^/K for many materials, which gives the error of density of the nuclear spin and resultant *T*_2_ on the order of only 0.1 to 1%, when the temperature changes between 0 K and 300 K, which does not affect the screening of materials for quantum coherence.

## Supplementary Material

Supplementary File

## Data Availability

CCE calculation codes, calculated datasets, and scripts used in materials exploration have been deposited in Zenodo at https://zenodo.org/record/6323098 (45) and Qresp (https://paperstack.uchicago.edu/paperdetails/62302ab3057dbbfb35b05d52?server=https%3A%2F%2Fpaperstack.uchicago.edu) (46).
