# Supplementary material for: Generalized scaling of spin qubit coherence in over 12,000 host materials
Source: Proc Natl Acad Sci U S A. 2022 Apr 6;119(15):e2121808119. doi: 10.1073/pnas.2121808119 (PMC9169712; doi:10.1073/pnas.2121808119)
Supplement: Supplementary File [file pnas.2121808119.sapp.pdf]

PNAS
www.pnas.org

Supplementary Information for
Generalized scaling of spin qubit coherence in over 12,000 host materials

Shun Kanai[1-5], F. Joseph Heremans[6,7], Hosung Seo[8], Gary Wolfowicz[6,7], Christopher P. Anderson[7,9], Sean E. Sullivan[6], Mykyta Onizhuk[10], Giulia Galli[10,6,7], David D. Awschalom[9,6,7,*], and Hideo Ohno[1,4,5,11,12]

[1]Laboratory for Nanoelectronics and Spintronics, Research Institute of Electrical Communication, Tohoku University, 2-1-1 Katahira, Aoba-ku, Sendai 980-8577, Japan
[2]PRESTO, Japan Science and Technology Agency, Kawaguchi 332-0012, Japan
[3]Division for the Establishment of Frontier Sciences of Organization for Advanced Studies at Tohoku University, Tohoku University, 2-1-1 Katahira, Aoba-ku, Sendai 980-8577, Japan
[4]Center for Science and Innovation in Spintronics, Tohoku University, 2-1-1 Katahira, Aoba-ku, Sendai 980-8577, Japan
[5]Center for Spintronics Research Network, Tohoku University, 2-1-1 Katahira, Aoba-ku, Sendai 980-8577, Japan
[6]Center for Molecular Engineering and Materials Science Division, Argonne National Laboratory, Lemont, IL 60439, USA
[7]Pritzker School of Molecular Engineering, University of Chicago, Chicago, IL 60637, USA
[8]Department of Physics and Department of Energy Systems Research, Ajou University, Suwon, Gyeonggi 16499, Republic of Korea
[9]Department of Physics, University of Chicago, Chicago, IL 60637, USA
[10]Department of Chemistry, University of Chicago, Chicago, IL 60637, USA
[11]Center for Innovative Integrated Electronic Systems, Tohoku University, 468-1 Aramaki Aza Aoba, Aoba-ku, Sendai 980-0845, Japan
[12]WPI-Advanced Institute for Materials Research, Tohoku University, 2-1-1 Katahira, Aoba-ku, Sendai 980-8577, Japan
David D. Awschalom
*Email: awsch@uchicago.edu

This PDF file includes:

Supplementary text
Figures S1 to S9
Table S1
SI References

Supplementary Information Text

Abstract

We offer a straightforward means to predict the electron spin's quantum coherence time in high quality, wide-bandgap solid-state systems at cryogenic temperatures, where the electron spin coherence time is well determined only by the effect of nuclear spins in the host materials. There, however, the coherence time still depends on the combination of material/experimental properties of the electron spin (*g*-factor, quantum number, zero-field splitting), the surrounding nuclear spin species (density, abundance, *g*-factor, quantum number), the host's crystal structure (lattice constant, symmetry), and the external magnetic field. In the following sections in this Supplemental Information, we show the way we disentangle these combinatorial factors based on the cluster correlation expansion calculations.

1. Spin Hamiltonian and density matrix

We consider the spin Hamiltonian $\mathcal{H}$ defined by

$$\mathcal{H} = \mathcal{H}_{\mathrm{S}} + \mathcal{H}_{\mathrm{B}} + \mathcal{H}_{\mathrm{S-B}}, \qquad \text{[S1]}$$

where $\mathcal{H}_{\mathrm{S}}$, $\mathcal{H}_{\mathrm{B}}$ are Hamiltonians for electron spin and nuclear spins, respectively, and $\mathcal{H}_{\mathrm{S-B}}$ indicates electron spin – nuclear spin interaction (1-3).

$$\mathcal{H}_{\mathrm{S}} = -g_{\mathrm{e}}\mu_{\mathrm{B}}BS_z, \qquad \text{[S2]}$$

$$\mathcal{H}_{\mathrm{B}} = -\sum_i g_i\mu_{\mathrm{N}}BI_{z,i} + \mathcal{H}_{\mathrm{n-n}}, \qquad \text{[S3]}$$

$$\begin{aligned}\mathcal{H}_{\mathrm{S-B}} &\approx \frac{\mu_0}{4\pi} g_{\mathrm{e}}\mu_{\mathrm{B}}\mu_{\mathrm{N}}\vec{S}\cdot\sum_i g_i\left[\frac{\vec{I}_i}{{r_i}^3} - \frac{3(\vec{I}_i\cdot\vec{r}_i)\vec{r}_i}{{r_i}^5}\right] \\ &\approx S_z\sum_i \vec{A}_i\cdot\vec{I}_i,\end{aligned} \qquad \text{[S4]}$$

where g_{e}, g_i, μ_{B}, μ_{N}, and μ_0 are the *g*-factor of electron, the *g*-factor of nuclear spin of nucleus i, Bohr magneton, nuclear magneton, and permeability of the vacuum, respectively. We set the magnetic field direction to be z direction. $\vec{r}_i$, r_i, B, $\vec{A}_i$, $\vec{S}$, S_z, $\vec{I}_i$, and $I_{z,i}$ are vectors from the electron spin to the nucleus i, $|\vec{r}_i|$, the magnetic field, the hyperfine field vector of nucleus i, the electron spin vector operator, z component of electron spin operator, the spin operator of nucleus i, and the z component of spin operator of nucleus i, respectively. $\mathcal{H}_{\mathrm{n-n}}$ is the Hamiltonian of the nuclear spin – nuclear spin interactions;

$$\mathcal{H}_{\mathrm{n-n}} = \frac{\mu_0}{4\pi}\mu_{\mathrm{N}}^2\sum_{\{i,j\}} g_ig_j\left[\frac{\vec{I}_i\cdot\vec{I}_j}{r_{ij}^3} - \frac{3(\vec{I}_i\cdot\vec{r}_{ij})(\vec{I}_j\cdot\vec{r}_{ij})}{r_{ij}^5}\right], \qquad \text{[S5]}$$

where $\vec{r}_{ij}$ is the vector from nucleus i to nucleus j, and $r_{ij} = |\vec{r}_{ij}|$. Two of the approximations in Eq. **S4** is valid when (1) the Fermi contact term is negligible with a localized electron spin center and dilute nuclear spins in the host, which is valid in most of the intrinsic and extrinsic defects in, *e.g.*, SiC and diamond, and (2) two of the electron spin states are separated on the order of GHz, *e.g.*, when one applies, for the $g_{\mathrm{e}} = 2$ defect, a magnetic field B larger than 30 mT, which is a standard

measurement condition for electron spin resonance measurements.

Under the secular approximation, the electron spin operator with $S > 1/2$ can be treated as the pseudo-spin. When we consider a generic magnetic dipole coherence $|m_S\rangle$: $|m_0 - 1/2\rangle \leftrightarrow |m_0 + 1/2\rangle$ (m_0: half integer), S_z is defined as a 2×2 matrix with the component of $\delta_{l,m}(m_0 \mp 1/2)$, where $\delta_{l,m}$ is Kronecker's delta. As with previous reports, we assume the maximum distance for the finite electron spin - nuclear spin (nuclear spin - nuclear spin) interactions as $r_{\mathrm{e-n}}$ ($r_{\mathrm{n-n}}$). The distances $r_{\mathrm{e-n}}$ and $r_{\mathrm{n-n}}$ depend on the magnetic field, the *g*-factor of electron spin, its quantum number, the *g*-factors of nuclear spins, their quantum numbers, their density, and compound's structure. We calculate the coherence time with the different limit lengths $r_{\mathrm{e-n}}$ and $r_{\mathrm{n-n}}$, which are optimized for each condition. Typical numbers of effective interacting nuclear spins within $r_{\mathrm{e-n}}$ are ~ 1,000 (1, 4).

2. Time evolution

Time evolution of the density matrix of the free induction decay (FID) and (Hahn echo) $\rho_{\mathrm{FID(Hahn)}}(t_{\mathrm{free}})$ is calculated by

$$\rho_{\mathrm{FID(Hahn)}}(t_{\mathrm{free}}) = \mathcal{U}_{\mathrm{FID(Hahn)}}(t_{\mathrm{free}})\rho(0)\mathcal{U}_{\mathrm{FID(Hahn)}}{}^{\dagger}(t_{\mathrm{free}}). \quad \text{[S6]}$$

We use the standard free induction decay (FID) propagator composed of $(\pi/2)_x$ pulse and free evolution for t_{free}, and Hahn echo propagator composed of $(\pi/2)_x$ pulse, free evolution for $t_{\mathrm{free}}/2$, π_x pulse, and free evolution for $t_{\mathrm{free}}/2$, as

$$\mathcal{U}_{\mathrm{FID}}(t_{\mathrm{free}}) = \exp\left(-i\frac{\mathcal{H}}{\hbar}t_{\mathrm{free}}\right)\exp\left(i\frac{\pi}{2}S_x\right). \quad \text{[S7]}$$

$$\mathcal{U}_{\mathrm{Hahn}}(t_{\mathrm{free}}) = \exp\left(-i\frac{\mathcal{H}}{\hbar}\frac{t_{\mathrm{free}}}{2}\right)\exp(i\pi S_x)\exp\left(-i\frac{\mathcal{H}}{\hbar}\frac{t_{\mathrm{free}}}{2}\right)\exp\left(i\frac{\pi}{2}S_x\right). \quad \text{[S8]}$$

The initial density matrix is taken to be $\rho_{\mathrm{FID(Hahn)}}(0) = \rho_{\mathrm{S}}(0)\otimes\rho_{\mathrm{B}}(0)$ using an electron spin projected density matrix $\rho_{\mathrm{S}}(0)$ to the z projection of initial spin $m_{\mathrm{S}} = m_0 - 1/2$ state

$$\rho_{\mathrm{S}}(0) = |m_0 - 1/2\rangle\langle m_0 - 1/2|, \quad \text{[S9]}$$

and a bath density matrix $\rho_{\mathrm{B}}(0)$

$$\rho_{\mathrm{B}}(0) = \sum_{\mathcal{J}} \mathcal{P}_{\mathcal{J}}|\mathcal{J}\rangle\langle\mathcal{J}| \quad \text{[S10]}$$

with $\mathcal{P}_{\mathcal{J}}$ being the probability of the nuclear state $|\mathcal{J}\rangle$. We assumed a fully thermalized nuclear spin bath, thus $\rho_{\mathrm{B}}(0)$ is the identity matrix. Hahn echo (FID) signal $\mathcal{L}(t_{\mathrm{free}})$ ($\mathcal{L}^*(t_{\mathrm{free}})$) is calculated by

$$\mathcal{L}(t_{\mathrm{free}})(\mathcal{L}^*(t_{\mathrm{free}})) = \frac{\mathrm{Tr}\left[\rho_{\mathrm{Hahn(FID)}}(t_{\mathrm{free}})S_+\right]}{\mathrm{Tr}\left[\rho_{\mathrm{Hahn(FID)}}(0)S_+\right]}, \quad \text{[S11]}$$

where S_+ is raising operator of electron spin (5).

3. Cluster correlation expansion (CCE) calculations

Figure S1 depicts the CCE scheme. The FID or Hahn-echo signals $\mathcal{L}^{\mathrm{CCE-1}}$ and $\mathcal{L}^{\mathrm{CCE-2}}$ obtained by first-, and second-order CCE (CCE-1 and CCE-2) calculations, respectively, are defined as (6)

$$\mathcal{L}^{\mathrm{CCE-1}} = \prod_i \mathcal{L}_i, \quad \text{[S12]}$$

$$\mathcal{L}^{\mathrm{CCE-2}} = \mathcal{L}^{\mathrm{CCE-1}} \prod_{\{i,j\}} \frac{\mathcal{L}_{i,j}}{\mathcal{L}_i \mathcal{L}_j}, \quad \text{[S13]}$$

where $\mathcal{L}_i$ ($\mathcal{L}_{i,j}$) is the coherence signals calculated with the central electron spin and the i-th nuclear spin (the electron spin and the i-th and j-th nuclear spins). It is known that for FID signal $\mathcal{L}^*(t_{\mathrm{free}})$, first order CCE well explains the characteristic time of the decay (7). We have confirmed that the effect including the three or higher body spin cluster interactions in Hahn-echo signal are negligible and $\mathcal{L}(t_{\mathrm{free}})$ converges with second-order CCE by the CCE-3 calculations on CeO_2, CeO_2, CaS, S, WS_2, and WO_3, which is consistent with the previous report on naturally isotopic diamond and 4*H*-SiC (1, 8).

4. Example CCE calculations

According to the generalized criteria consolidated by Weber, Koehl, Varley *et al.* (WKV criteria) (9), materials with a wide bandgap, small spin-orbit coupling, spinful nuclear spin free lattice, and availability of high-quality bulk or thin film single crystal is preferrable for the host materials of spin defect (10). In addition to typical materials such as SiC or diamond, here we choose oxides as a model host system, because many of them meet the criteria: a bandgap large enough to include isolated color centers optically accessible with visible and/or near-infrared lasers, small spin-orbit couplings due to small atomic number of anion (O), and a low spinful nuclear spin density in a naturally abundant anion (^{17}O 0.038%). In addition, they are often easy to fabricate into thin films or bulk single crystals, and compatible with established nanofabrication techniques.

Figure S2 shows the calculated FID and Hahn-echo signal as a function of the total free evolution time t_{free} in typical widegap oxides as well as diamond, Si, and 4*H*-SiC. We assume the electron spin with S = 1/2 and its *g*-factor g_{e} = 2 under B = 5 T. The FID signal is simulated by using CCE-1, and the Hahn-echo signal is calculated by using CCE-2.

The FID is critically affected by the slow magnetic noise from the nuclear spins to the electron spin center. Under the strong magnetic field, FID signal for an S = 1/2 electron spin and surrounding I = 1/2 spins with a density of n_i is approximated by $e^{-t_{\mathrm{free}}/T_2^*}$, where T_2^* is the inhomogeneous dephasing time, and $T_2^* \propto (n_i g_e g_i)^{-1}$ (7). Considering the definition of the CCE-1 (Eq. **S12**), a compound's FID signal is expressed as $\prod_i e^{-t_{\mathrm{free}}/T_{2,i}^*}$ with the effective dephasing time of the nucleus i ($T_{2,i}^*$) and $T_2^* = \left[\sum_i \left(T_{2,i}^*\right)^{-1}\right]^{-1}$, indicating the nucleus with the shortest effective dephasing time, *i.e.*, the nucleus with the largest product of the nuclear spin density and the *g*-factor $n_i g_i$, dominates a compound's dephasing time T_2^*.

For the Hahn-echo signal, with B = 5 T, the collapse and revival that is generally seen at small B (1, 11) is suppressed, and the homogeneous dephasing time (T_2) is also fully saturated with respect to B. There, the slow magnetic noise contributions are refocused by a π pulse, and the decoherence mainly originates from the interactions between nuclear spins. T_2 is obtained by fitting the coherence function $\mathcal{L}(t_{\mathrm{free}})$ with a stretched decay function $e^{-(t_{\mathrm{free}}/T_2)^{\eta}}$, where η is the stretching exponent, which typically has a value between 2 and 3 (12). We find many oxides with natural isotope abundance with a larger T_2 than that of SiC. In particular, in the oxides CeO_2 (53.5(13) ms) and CaO (37.2(71) ms), there is either no or extremely low concentrations of spinful nuclei in both the anions and cations (Ce: no spinful nuclear spin; Ca: ^{43}Ca with spin 7/2 and 0.14%

natural abundance), and offer T_2 values about 40 times greater than SiC. SiO_2 (α-quartz, 3.42(15) ms) and ZnO (wurtzite, 2.33(28) ms) are also predicted to possess milliseconds-long T_2, while MgO (0.90(8) ms) shows almost the same T_2 as that of diamond (0.97(12) ms).

The similar trends of the materials' T_2 and T_2^* are obtained as shown in the Table S1, implying that T_2 is also critically affected by the shortest effective coherence time of the nucleus i ($T_{2,i}$). In contrast to the FID signal, however, because the spin dynamics of the Hahn echo is dominated by the interactions between nuclear spins, the signal cannot be decomposed/simplified in terms of scaling the Ramsey time without assumptions.

5. Effect of quadrupole interaction

The quadrupole interaction is defined as

$$\mathcal{H}_{\mathrm{Q}} = \frac{e^2qQ}{4I(2I-1)}\left[3I_z^2 - \vec{I}^2 + \epsilon_{\mathrm{as}}\left(I_x^2 - I_y^2\right)\right], \quad \text{[S14]}$$

where e is the elementary charge, $eq \equiv \partial^2V/\partial z^2$, V is the electric potential, Q is the electric quadrupole moment, ϵ_{as} is the asymmetry parameter of the electric field gradient defined as $\epsilon_{\mathrm{as}} = (\partial^2V/\partial x^2 - \partial^2V/\partial y^2)/(\partial^2V/\partial z^2)$, and I is nuclear spin quantum number (I > 1/2).

In cubic compounds, *e.g.*, CeO_2, CaO, and CaS, there are no quadrupole interactions due to the central inversion symmetry of V. In the other symmetry group materials, however, $\mathcal{H}_{\mathrm{Q}}$ can nontrivially change the dynamics of the nuclear spin bath.

In order to quantitatively estimate the effect of the quadrupole interaction, we calculate coherence times of the several compounds with finite $\mathcal{H}_{\mathrm{Q}}$. For example, Fig. S3 shows the coherence time of the naturally abundant WS_2 (W: ^{183}W with spin 1/2 and 14% natural abundance; S: ^{33}S with spin 3/2 and 0.75% natural abundance). We assume quadrupole tensors for the nuclear spins in the vicinity of the defect to be the same as in the bulk material. This assumption is justified for the very low concentration of ^{33}S spins. Each ^{33}S has C_{3V} symmetry, and the quadrupole tensor has only an axial component (P_{zz}) parallel to the *c*-axis of the crystal (parallel to z and B) with ϵ_{as} = 0. P_{zz} dependence of T_2 shows that the increase of P_{zz} increases T_2 up to 30%, and T_2 saturates at P_{zz} larger than dipole-dipole interaction (~ Hz).

The quadrupole couplings effectively decouple the different transitions of nuclear spins, *e.g.* for two nuclear spin with I = 1 the transition of $|-1+1\rangle \leftrightarrow |00\rangle$ is not allowed due to the different energy splitting between +1/0 and 0/-1 energy levels. When they are fully decoupled with quadrupole interaction much larger than dipole-dipole interaction, the actual amplitude of quadrupole coupling becomes irrelevant, which is observed in the Fig. S3.

In the CCE calculations other than this section, we have ignored the quadrupole moment, which is suitable for estimating a lower bound of the coherence time of the defect in the given materials.

6. Decoupling field

In this section, we estimate the decoupling magnetic field (B_{dec}), under which heteronuclear spin baths in a compound are decoupled. The envelope of the Hahn-echo signal is critically affected by the dipole-dipole interactions between the nuclear spins. The dipole-dipole interactions between the heteronuclear spins are characterized by two factors, Ω and Δ, where Ω indicates the dipole-dipole interactions between nucleus i and j, which is given by Eq. **S5**. Δ indicates the energy splitting between two levels interacting through $I_{+,i}I_{-,j} + I_{-,i}I_{+,j}$ due to the different Zeeman splitting with different nuclear spin g-factors between nucleus in addition to the dipole-dipole interaction between them with $I_{\pm,i}$ being the ladder operator of spin in nucleus i given by Eq. **S3** and the

hyperfine interaction given by Eq. **S4**. When $\Delta \gg \Omega$, heteronuclear spin baths are decoupled.

Let us consider the nuclear spin for the spin-1/2 nucleus. When we accept the secular approximation, the Hamiltonian used in the CCE-2 can be written by

$$H = \begin{pmatrix} H_+ & O \\ O & H_- \end{pmatrix}, \tag{S15}$$

where H_+ and H_- are the subset Hamiltonians for the electron states $|m_S^+\rangle$, and $|m_S^-\rangle$, respectively, which interact with the nearby nuclear spin pairs of nucleus i and j, positioned at $\overrightarrow{r_k} = r_k(\sin\theta_k \cos\varphi_k, \sin\theta_k \sin\varphi_k, \cos\theta_k)$ $(k = \{i,j\})$. H_+ is

$$H_{11} = \begin{pmatrix} A + B_i + B_j + D_i + D_j + J & E_j^- + L^- & E_i^- + L^- & P^- \\ E_j^+ + L^+ & A + B_i - B_j + D_i - D_j - J & -J & E_i^- - L^- \\ E_i^+ + L^+ & -J & A - B_i + B_j - D_i + D_j - J & E_j^- - L^- \\ P^+ & E_i^+ - L^+ & E_i^+ - L^+ & A - B_i - B_j - D_i - D_j + J \end{pmatrix}, \tag{S16}$$

where $A = -g_e\mu_B B$, $B_k = -g_k\mu_N B$, $D_k = -\frac{\mu_0}{4\pi} g_e\mu_B\mu_N \frac{g_k}{r_k{}^3}(3\cos^2\theta_k - 1)$, $E_k^\pm = -\frac{\mu_0}{4\pi} g_e\mu_B\mu_N \frac{g_k}{r_k{}^3} \cdot 3\sin\theta_k \cos\theta_k\, e^{\pm i\varphi_k}$, $J = -\frac{\mu_0}{4\pi r_{ij}^3}\mu_N^2 g_i g_j(3\cos^2\theta_{ij} - 1)$, $L^\pm = -\frac{\mu_0}{4\pi r_{ij}^3}\mu_N^2 g_i g_j \cdot 3\sin\theta_{12}\cos\theta_{12}\, e^{\pm i\varphi_{12}}$, $P^\pm = -\frac{\mu_0}{4\pi r_{ij}^3}\mu_N^2 g_i g_j \cdot 3\sin^2\theta_{ij}\, e^{\pm 2i\varphi_{ij}}$, with $\overrightarrow{r_{ij}} = r_{ij}(\sin\theta_{ij}\cos\varphi_{ij}, \sin\theta_{ij}\sin\varphi_{ij}, \cos\theta_{ij})$. Previous reports revealed that the terms from $I_{z,i}I_{z,j}$ and spin flip-flop $I_{+,i}I_{-,j} + I_{-,i}I_{+,j}$ dominate the decoherence (1). The subset of H_+ for $|\uparrow\downarrow\rangle$ and $|\downarrow\uparrow\rangle$ states is

$$H'_+ = \begin{pmatrix} A + \Delta B + \Delta D - J & -J \\ -J & A - \Delta B - \Delta D - J \end{pmatrix}, \tag{S17}$$

where difference of the eigenstates is $\delta = 2\sqrt{(\Delta B + \Delta D)^2 + J^2}$, and the transition frequency $\Omega = J$, where $\Delta B \equiv B_i - B_j$ and $\Delta D \equiv D_i - D_j$. The decoupling condition $\Delta \gg \Omega$ gives

$$\begin{aligned} \mu_N B_{dec}(g_i - g_j) + \frac{\mu_0}{4\pi} g_e\mu_B\mu_N \left[\frac{g_i}{r_i{}^3}(1 - 3\cos^2\theta_i) - \frac{g_j}{r_j{}^3}(1 - 3\cos^2\theta_j)\right] \\ \gg \frac{\mu_0}{8\pi r_{ij}^3}\mu_N^2 g_i g_j(3\cos^2\theta_{ij} - 1). \end{aligned} \tag{S18}$$

For most cases, the second term with the difference of hyperfine interactions is dominated by the difference of the g-factors because the largest contribution for B_{dec} is given by the nearest-neighbor heterogeneous nucleus. Using the distance (l) of the nearest-neighbor nucleus $r_{ij} = l \ll r_i, r_j$, we obtain the upper limit of the B_{dec} as

$$B_{dec} = \frac{\mu_0}{4\pi}\mu_N \frac{1}{l^3}\frac{g_i g_j}{g_i - g_j}. \tag{S19}$$

B_{dec} for each element is listed in the Table S1. In SiC, for example, l = 1.33 Å, $g_{^{29}Si}$ = –1.11, and $g_{^{13}C}$ = +1.40 give B_{dec} = 0.13 mT. Using CCE calculations, Seo *et al.*, have numerically shown that B = 30 mT decouples heteronuclear spin baths in SiC even when the difference of the nuclear spin g-factor values (Δg) is decreased to 0.021 with keeping l = 1.33 Å (*cf.* in SiC, Δg = 2.51). This Δg and l values are relatively small in the compounds. In this condition, B_{dec} in Eq. **S20** gives 20 mT. In experiments, B up to 300 mT ~ 1 T is achievable with a standard yoke magnet. The decoupling field B_{dec} is proportional to $1/l^3\Delta g$, suggesting the heteronuclear spin baths are decoupled in most of the experimental conditions and materials.

As example systems, let us consider the oxides and sulfides. The ionic radius of the O^{2-} is 0.14 nm at minimum, which is the lowest limit of l for a material, and for the worst case among all isotopes, Δg = 0.024 for ^{9}Be gives B_{dec} ~5 mT. For sulfides, with the same means, the largest B_{dec} is given by ^{189}Os with Δg = 0.011, as ~3 mT.

7. Stretching exponent

In general, a Hahn-echo signal is fitted by the decay function $e^{-(t_{\mathrm{free}}/T_2)^{\eta}}$ with a stretching exponent η between 2-3 (1). The stretching exponent depends on the bath conditions, *e.g.*, the nuclear spin density (13) When the heteronuclear spin baths are decoupled, a compound's Hahn-echo signal $\mathcal{L}(t_{\mathrm{free}})$ is decomposed as $\prod_i \mathcal{L}_i(t_{\mathrm{free}})$ according to the definition of CCE-2 (Eqs. **S12** and **S13**) with $\mathcal{L}_i(t_{\mathrm{free}})$ being the Hahn-echo signal of the bath composed of nucleus i. Thus, the relation $e^{-(t_{\mathrm{free}}/T_2)^{\eta}} = \prod_i e^{-(t_{\mathrm{free}}/T_{2,i})^{\eta_i}}$, where $T_{2,i}$ and η_i are T_2 and η of for $\mathcal{L}_i(t_{\mathrm{free}})$, respectively, gives $\sum_i (T_2/T_{2,i})^{-\eta_i} = 1$. For most cases, $T_{2,i}$ and $T_{2,j}$ differ by several orders of magnitude, and one of the nucleus' T_2 dominates the compound's T_2. We find this T_2 is well approximated by

$$T_2 \approx \left(\sum_i T_{2,i}{}^{-\eta_i}\right)^{-\frac{1}{\eta'}}, \qquad \text{[S20]}$$

with η_i and η' assumed to be 2. Figure S4 shows the error of T_2 in binary compound with nucleus i and j between T_2 obtained by Eq. **S20** with η_i = η' = 2 and the exact T_2 as a function of ratio of $T_{2,i}$. The maximum T_2 errors among $2 < \eta_i \neq \eta_i < 3$ are plotted. For example, when $T_{2,j} = T_{2,i}/10$ ($T_{2,j} = T_{2,i}/3$), T_2 obtained by Eq. **S20** varies from the exact value by 0.44% (4.0%) at most.

8. Crystal structure and nuclear spin density dependences

Here we elucidate the Hahn echo with the different crystal structures. First, we see the lattice constant dependence. Figure S6a shows the ^{13}C abundance dependence of the T_2 in the diamond with the different lattice constants. Figure S6b shows the same T_2 replotted by the ^{13}C density ($n_{^{13}\mathrm{C}}$). S = 1/2 and g_{e} = 2 are assumed and B = 5 T is applied along the [111] (parallel to the C-C bonds) direction of the diamond lattice. Figures show that regardless of the different lattice constants, T_2 at $n_{^{13}\mathrm{C}}$ < 10^{21} cm^{-3} is well fitted by the power law $T_2 \propto n_{^{13}\mathrm{C}}^{\alpha}$. The exponent α does not change with lattice constant, and takes α = –1.008(13), which is consistent with previous reports based on numerical and analytical calculations suggesting α = –1 (1, 4). When one increases the density, the anisotropy of the dipole-dipole interactions appears and enhances T_2 (3, 4, 14, 15).

Most importantly, as shown in the main Fig. 2A, the scaling exponent and coefficient do not depend on the crystal structure as well. In addition, we calculate the T_2 for the baths with different nucleus and all of them obey the same exponent on the nuclear spin density (n_i) as

$$T_{2,i} = a(g_i, I_i, g_{\mathrm{e}}, S) n_i^{-1} \qquad \text{[S21]}$$

at n_i < 10^{21} cm^{-3} with the nucleus specific coefficient a.

In SiC, various intrinsic and extrinsic defects in several crystalline polytypes (3*C*, 4*H*, 6*H*, *etc*…) are utilized to achieve various functionalities (16, 17). Their optical properties drastically vary with the defect (site, and atom), and host (crystal structure, crystalline quality), while their coherence times in Hahn-echo signal around one millisecond have not been reported to change with the polytype (18). This is consistent with the facts that Si and C baths are decoupled in SiC, and $T_{2,i}$ is

governed by the spin density n_i and coefficient a_i independent on the crystalline structure or the defect type. The same phenomenon happens when we calculate SiO_2 with different polytypes (α-quartz, β-quartz, α-cristobalite, β-cristobalite, *etc*…). Those phenomena imply the existence of an "amorphous limit", where the effects of dilute nuclear spins are independent of the crystalline symmetry or the anisotropy and leading to our elucidation of the coherence times with the different crystal structures and the nuclear spin densities as above.

9. Generalized scaling of quantum coherences

In order to determine the coefficient $a(g_i, I_i, g_\mathrm{e}, S)$, we have calculated the Hahn-echo signal for all the stable species with S = 1/2 and g_e = 2. We adopt the crystalline structure and the lattice constant of the most common stable form of each element, and change their nuclear spin abundances to meet n_i = 1×10^{20} cm^{-3}. As in the main text, we define $a_i(g_i, I_i) \equiv a(g_i, I_i, g_\mathrm{e} = 2, S = 1/2)$. From the obtained $T_{2,i}$, we calculate $a_i = T_{2,i} n_i$ as shown in Fig. 2B.

Then, $a_i(g_i, I_i)$ is fitted by $b(I_i) g_i^{\beta(I_i)}$ for the different I_i. As shown in Fig. 2C, we find $\beta(I_i)$ is independent of I_i, thus, independent of nucleus i, and takes −1.64(7), which is in consistent with the theoretically obtained value −13/8 for the S = 1 defects with I_i = 1/2 baths. $b(I_i)$ changes with I_i, and is well fitted by $c \cdot I_i^\chi$ with χ = −1.10(3) and c = 1.46(4)×10^{18} cm^{-3} s. Thus, under S = 1/2 and g_e = 2, we conclude $T_{2,i}$ is scaled as (n_i in cm^{-3})

$$\begin{aligned} T_{2,i} &= a(g_i, I_i) \cdot n_i^\alpha \\ &= b(I_i) \cdot n_i^\alpha g_i^\beta \\ &= c \cdot n_i^\alpha g_i^\beta I_i^\chi \\ &= 1.46(4) \times 10^{18} \cdot n_i^{-1} g_i^{-1.64(7)} I_i^{-1.10(3)} \ (\mathrm{s}), \end{aligned} \qquad \text{[S22]}$$

and Eqs. **S21** and **S22** give each compound's T_2 as (n_i in cm^{-3}, S = 1/2, and g_e = 2)

$$T_2 \approx \frac{1.5 \times 10^{18}}{\sqrt{\sum_i n_i^2 g_i^{3.3} I_i^{2.2}}} \ (\mathrm{s}). \qquad \text{[S23]}$$

We repeat the same procedure above for other S and g_e defects. For S > 1/2 centers, a two-level system can be assigned to a given electron spin transition, acting similarly but not equivalently to S = 1/2 under the secular approximation. For S = 1, for example, T_2 is shown to be ~10% longer than that with S = 1/2 through CCE calculations (1). We consider a generic magnetic dipole transition $|m_\mathrm{S}\rangle$: $|m_0 - 1/2\rangle \leftrightarrow |m_0 + 1/2\rangle$ (m_0: half integer) for m_0 = 0 to 2, corresponding to S = 1/2 to 5/2 defects. Note that transitions of the same m_0 with different S, *e.g.*, $|-1/2\rangle \leftrightarrow |+1/2\rangle$ transitions in S = 1/2 and S = 3/2 centers, give the same T_2 because their spin sublevels are the same.

Figures S7 and S8 summarize the $a(g_i, I_i, g_\mathrm{e}, m_0)$, $b(I_i, g_\mathrm{e}, m_0)$, and $\beta(I_i, g_\mathrm{e}, m_0)$. For all (g_e, m_0) conditions, as shown in Fig. S7, $a(g_i, I_i, g_\mathrm{e}, m_0)$ is well fitted by $b(I_i, g_\mathrm{e}, m_0) \cdot g_i^{\beta(I_i, g_\mathrm{e}, m_0)}$. Furthermore, Fig. S8 shows that β is independent of (I_i, g_e, m_0) and takes constant value ~ −1.6, again which is in good agreement with the theoretically obtained value −13/8 for S = 1 and I = 1/2. Figure S8 also shows that $b(I_i, g_\mathrm{e}, m_0)$ is well fitted by $c(g_\mathrm{e}, m_0) I_i^{\chi(g_\mathrm{e}, m_0)}$, where the coefficient $c(g_\mathrm{e}, m_0)$ and the exponent $\chi(g_\mathrm{e}, m_0)$ are shown in Figs. S9a and b, respectively. χ does not depend on the g_e. χ takes the same value for m_0 = 0 ($|m_\mathrm{S}\rangle$: $|-1/2\rangle \leftrightarrow |+1/2\rangle$ transition) and m_0 = 1/2 ($|m_\mathrm{S}\rangle$: $|0\rangle \leftrightarrow |\pm 1\rangle$ transitions) of about −1.1 ~ −1.0, while for the case m_0 = 1 ($|m_\mathrm{S}\rangle$: $|+3/2\rangle \leftrightarrow |+1/2\rangle$ transition) χ takes smaller values. As shown in Fig. S9a, c is well fitted by $d(m_0) g_\mathrm{e}^{\delta(m_0)}$. The

coefficient $d(m_0)$ and the exponent $\delta(m_0)$ are plotted in Figs. S9c and d, respectively. The exponent δ is about −0.4, which is close to the value −3/8 analytically obtained for S = 1 and I = 1/2. $d(m_0)$ increases with increasing m_0 and is fitted by a polynomial function $d = 1.9 \times 10^{18}(1 + 0.57m_0^2)$ cm^{-3} s.

Overall, surprisingly, the factors determining the $T_{2,i}$ (g_i, I_i, g_e, m_0) are well separated except for m_0 by the form of scaling with individual parameters as (n_i in cm^{-3})

$$T_{2,i} = d \cdot n_i^\alpha g_i^\beta I_i^\chi g_e^\delta = \begin{cases} 1.9 \times 10^{18} \cdot n_i^{-1} g_i^{-1.6} I_i^{-1.1} g_e^{-0.39}\ (\mathrm{s}) & \text{for } m_0 = 0 \\ 2.2 \times 10^{18} \cdot n_i^{-1} g_i^{-1.6} I_i^{-1.1} g_e^{-0.39}\ (\mathrm{s}) & \text{for } m_0 = 1/2. \\ 3.0 \times 10^{18} \cdot n_i^{-1} g_i^{-1.6} I_i^{-1.2} g_e^{-0.38}\ (\mathrm{s}) & \text{for } m_0 = 1 \end{cases} \quad [S24]$$

10. Increase of the coherence time with m_0

When one changes m_0, only the hyperfine interactions change in the spin Hamiltonian in Eq. **S1**. The hyperfine interaction plays two important roles in the central spin decoherence: (1) The nuclear spins undergoing flip-flop transitions induce a fluctuation in the transition frequency of the electron spin through the hyperfine interaction. (2) The hyperfine interaction acts as a position-dependent effective magnetic field on the nuclear spins, shifting their levels in energy in addition to the Zeeman shift. In our study, we consider the single electron spin transition (*i.e.* Δm_S = 1). Thus, for a given magnetic noise level in the nuclear spin bath, the fluctuation induced in the electron spin transition frequency may be the same regardless of the m_0 value. However, the strength of the position-dependent effective field due to the hyperfine interaction becomes larger with an increase of $|m_S|$ ($\equiv |m_0 \pm 1/2|$).

The magnetic noise in the central spin created by the nuclear flip-flop transitions can be well understood by using the pseudo-spin model (1). For two homo-nuclear spins (I_i and I_j) in the presence of large external magnetic field, the only relevant transitions occur between $|\uparrow\downarrow\rangle$ and $|\downarrow\uparrow\rangle$ states, which can be modelled as a pseudo-spin. The flip-flop transition is mediated by the dipolar interaction at a rate $\Omega = \langle\uparrow\downarrow|\mathcal{H}_{\mathrm{n-n}}|\downarrow\uparrow\rangle$, where $\mathcal{H}_{\mathrm{n-n}}$ is the interaction Hamiltonian defined in Eq. **S5**. The transition rate is typically about a few Hz to tens of Hz in a dilute nuclear spin bath. The level splitting between the two is determined by the hyperfine interaction, which is typically about kHz in a dilute nuclear spin bath; $\Delta = m_S(A_{z,j} - A_{z,i})$. While the rate Ω is independent of m_S, the frequency detuning Δ increases with an increase of m_S, suppressing the flip-flop transitions. Therefore, we see that T_2 can increase as m_0 increases, which is consistent with our finding that d increases with increasing m_0.

11. Example of predicted material / functionality

Beyond considerations of the nuclear spin environment, the spin coherence properties of individual materials should ultimately be evaluated on a case-by-case basis; FeO (#2, 36 ms), for example, is predicted to have the second longest T_2 explained by the fact that Fe has a quite long T_2 as shown in Table I due to a small nuclear spin *g*-factor, a low natural abundance of spinful isotope, and a small I_{Fe}. On the other hand, FeO is antiferromagnetic with a Néel temperature of 198 K, which likely has a critical effect on coherence, *i.e.*, the effect of macroscopic magnetic texture of electron spins except below the cryogenic temperature and above the Néel temperature (19). This new path between the electron spin of defect center and mesoscopic electron also might offer a new controllability of spin center by, for example, switching the interaction between them through electric field control of the magnetic phase (20, 21).

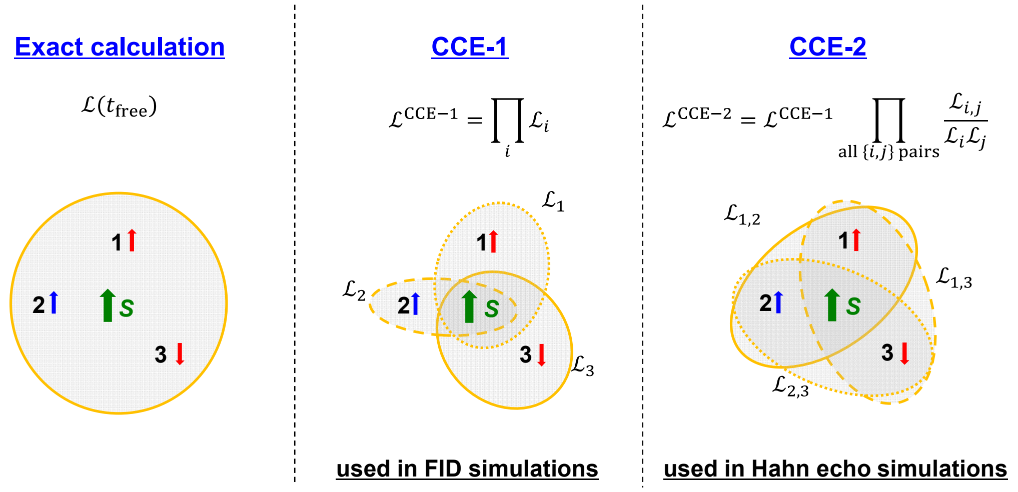

Fig. S1 | Cluster correlation expansion. Schematics of the effective Hamiltonians in cluster correlation expansion (CCE) with different orders.

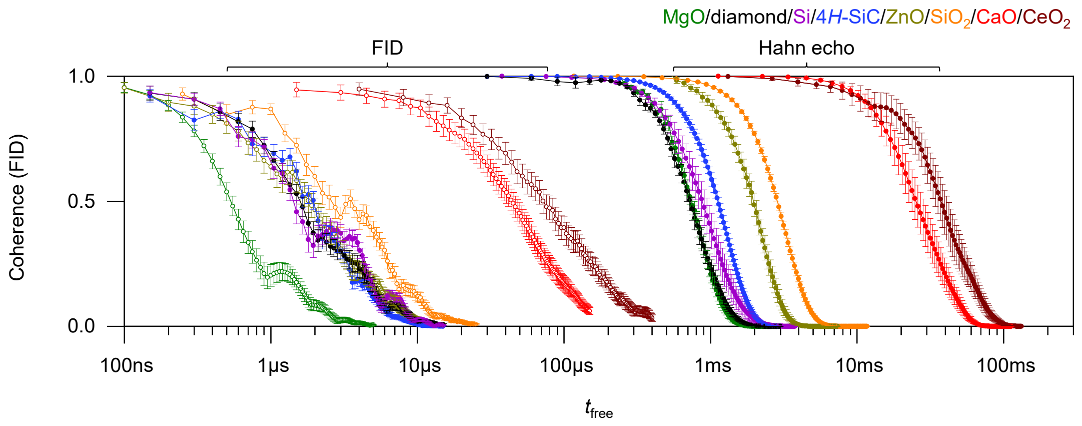

Fig. S2 | Coherence time of oxides and other spin defect host. Free induction decay (FID) signal calculated by CCE-1 and Hahn-echo signal calculated by CCE-2 under external magnetic field with B = 5 T.

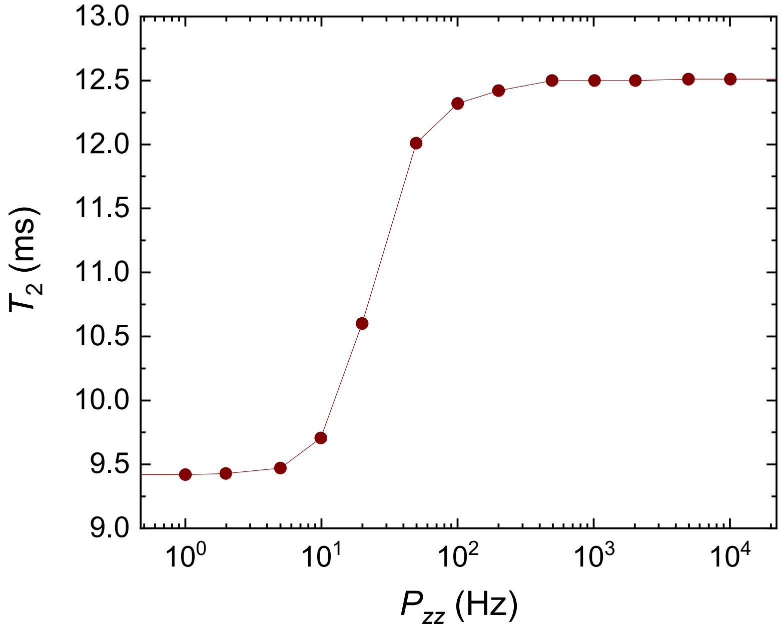

Fig. S3 | Effect of the quadrupole moment on the coherence time. Simulated coherence time (T_2) of naturally abundant WS_2 as a function of the axial component of quadrupole interaction (P_{zz}) parallel to the *c*-axis. External magnetic field B = 5 T is applied along the *z*-axis.

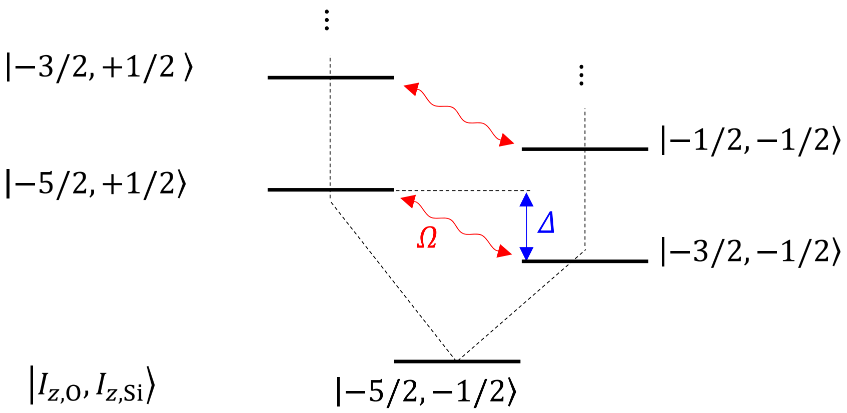

Fig. S4 | Dipole-dipole interaction between nuclear spins. Schematics of the energy states in SiO_2 with one ^{17}O (g_i = –0.758) and one ^{29}Si (g_i = –1.11) nucleus under finite dipole-dipole interactions Ω. With different nuclear spin Zeeman effect due to the different *g*-factors, each state interacting with the flip-flop transitions split by Δ.

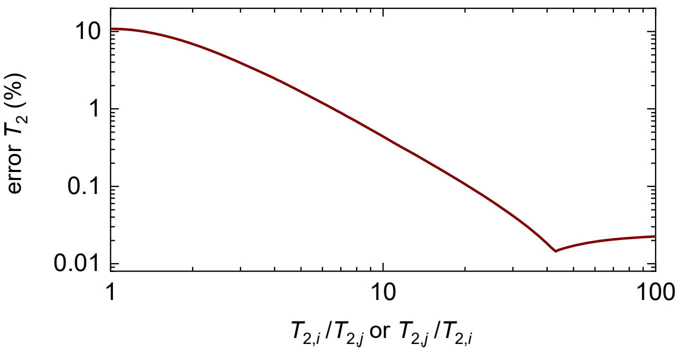

Fig. S5 | Error of approximation under constant stretching exponent. Error of T_2 in binary compound with nucleus i and j between T_2 obtained by Eq. **S20** with η_i = η' = 2 and the exact T_2 as a function of ratio of $T_{2,i}$. The maximum T_2 error among all (η_i, η_j) pairs in $2 \leq \eta_i, \eta_j \leq 3$ are plotted.

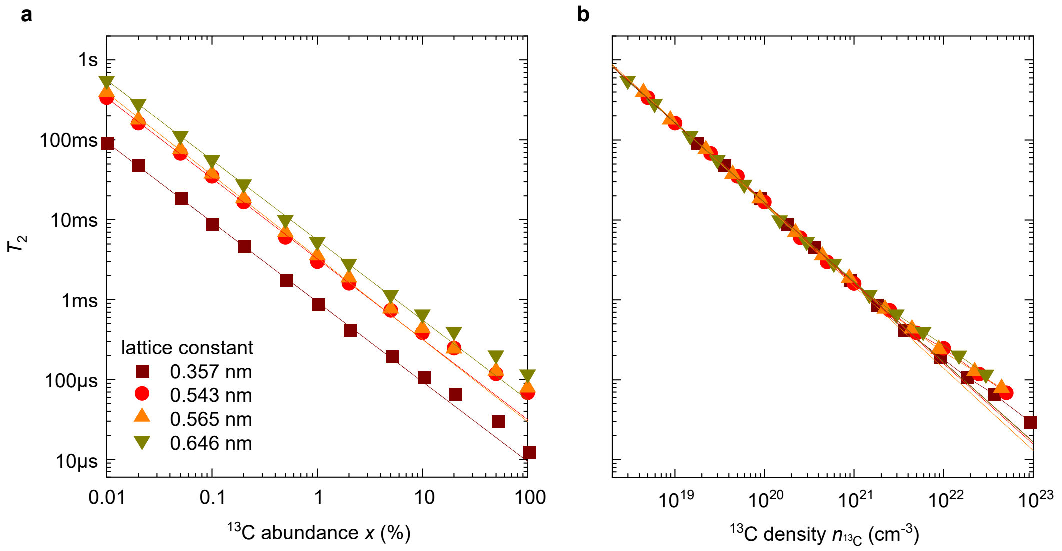

Fig. S6 | Lattice constant dependence of T_2 in diamond. a, T_2 calculated for the diamond with the different lattice constants and ^{13}C abundances. The lines show the power-law fits. The lattice constants of the diamond structure are chosen to be those of diamond (0.357 nm), Si (0.543 nm), Ge(0.565 nm), and α-Sn (0.646 nm). **b**, The same T_2 as (**a**) plotted as a function of ^{13}C density ($n_{^{13}C}$).

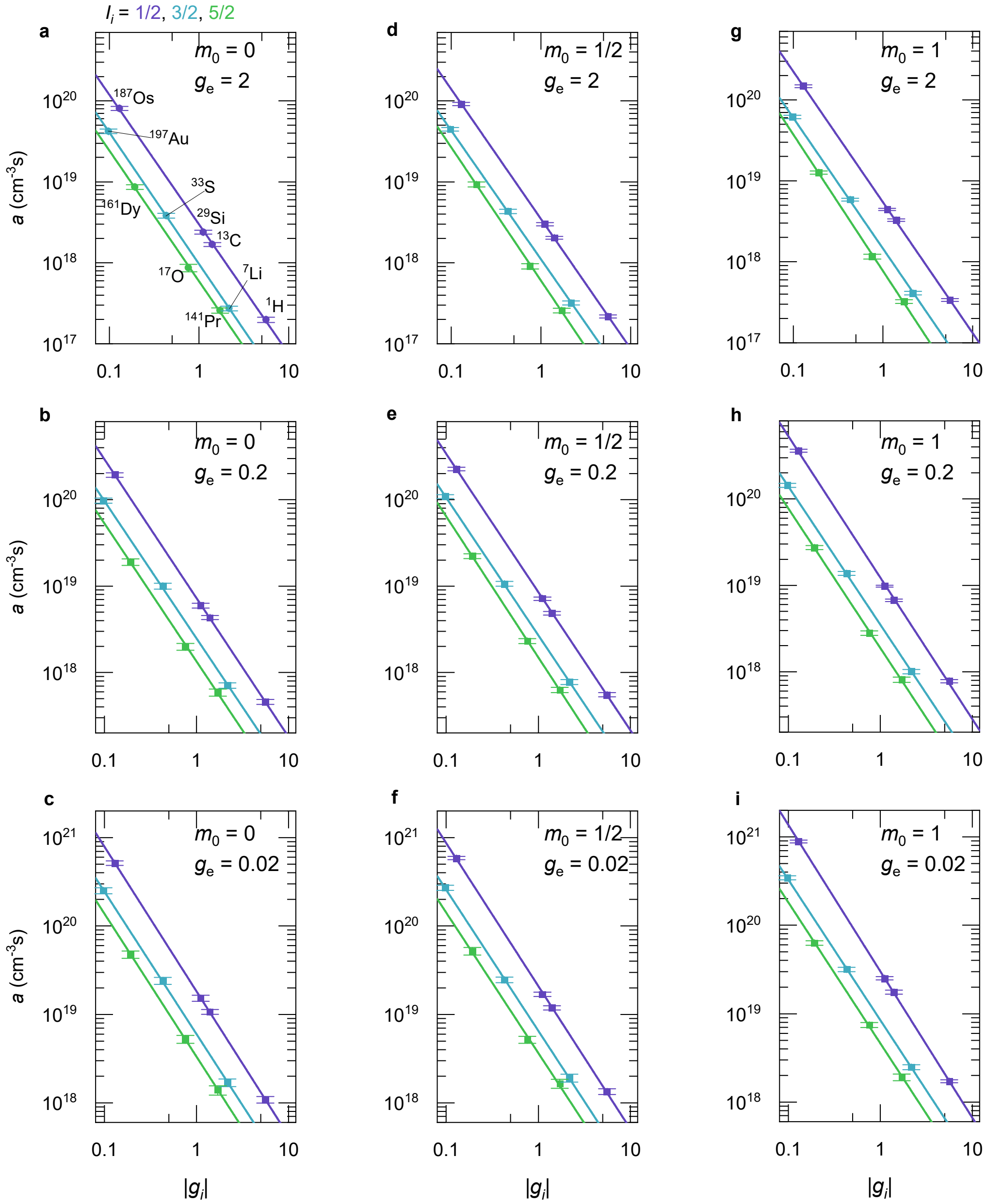

Fig. S7 | Scaling with the nuclear spin *g*-factor. Coefficient a calculated with nuclear spin density n_i = 1.0×10^{20} cm^{-3} as a function of absolute value of nuclear spin *g*-factor $|g_i|$. Lines are exponential fits $a = b|g_i|^{\beta}$ on the different half-integer-I_i spins with different electron spin transition sub levels ($|m_S\rangle = |m_0 \pm 1/2\rangle$) and electron spin *g*-factors (g_e). Error bars indicate the sample standard deviation obtained by the simulation for different crystal configurations.

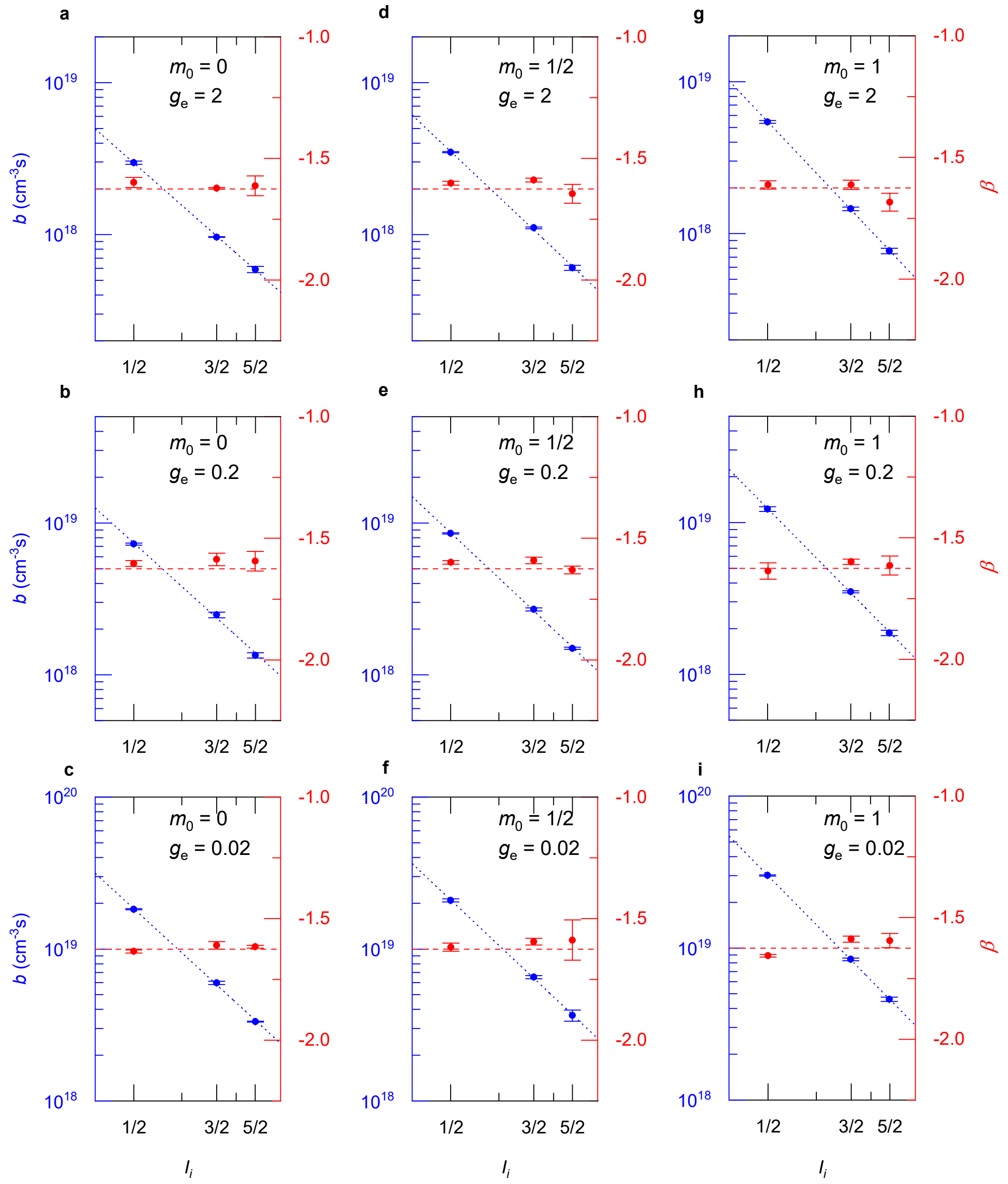

Fig. S8 | Scaling with the nuclear spin quantum number. Coefficient b (left axes, blue) and exponent β (right axes, red) as a function of nuclear spin quantum number I_i obtained by fitting to Fig. S7 for different electron spin transition sub levels ($|m_S\rangle = |m_0 \pm 1/2\rangle$) and electron spin g-factors (g_e). Blue dashed lines are power fits $b = cI_i{}^{\chi}$ and the red dashed lines are constant value −13/8 as explained in the main text. Error bars indicate the standard deviation of the fittings.

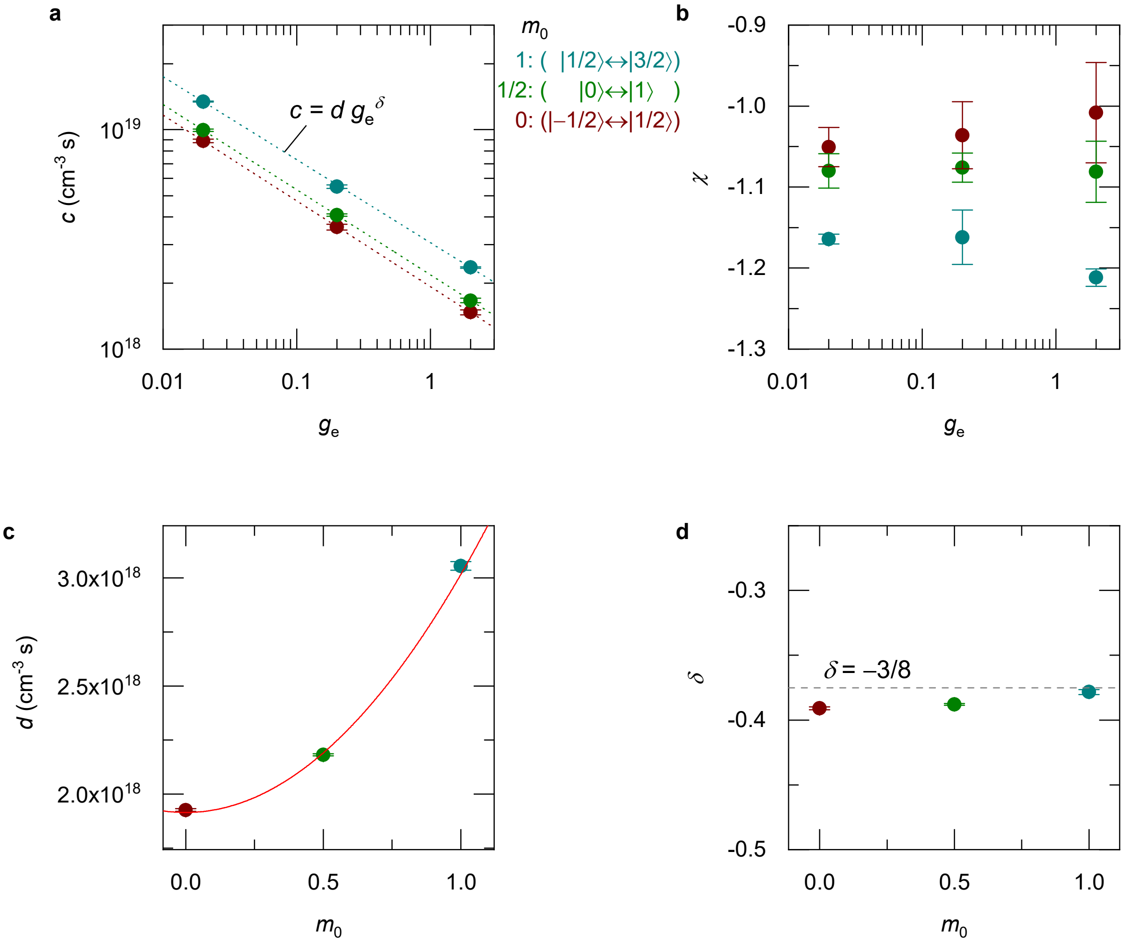

Fig. S9 | Scaling with electron spin *g*-factor. a, Coefficient c *vs.* electron spin *g*-factor (g_{e}) and **b,** exponent χ *vs.* g_{e} with the different electron spin transition sub levels ($|m_S\rangle = |m_0 \pm 1/2\rangle$) obtained by fitting to Fig. S8. Dotted lines are exponential fits $c = dg_{\mathrm{e}}^{\delta}$. **c,** Coefficient d *vs.* m_0. The solid line shows the polynomial fit as explained in the main text. **d,** Exponent δ *vs.* m_0. The dashed line is constant value –3/8 as explained in the main text. Error bars indicate the standard deviation of the fittings.

Table S1 | Decoherence time and decoupling fields. Inhomogeneous dephasing time (T_2^*) simulated by FID, and homogeneous dephasing time (T_2) and stretching exponent (η) simulated by Hahn echo. Values are ensemble average and errors show standard deviation. Decoupling fields (B_{dec}) for heteronuclear spin baths calculated with Eq. **S20**.

**Material**	T_2^* **(ms)**	T_2 **(ms)**	η	B_{dec} **(mT)**
diamond	4.2(12)	0.97(12)	2.64(29)	-
SiC (4*H*)	5.3(16)	1.35(9)	2.80(14)	0.13
Si	4.9(17)	1.24(23)	2.30(33)	-
CeO_2	179(128)	53.5(13)	2.80(53)	-
CaO	77(33)	37.2(71)	2.84(49)	0.027
SiO_2 (α-quartz)	8.5(29)	3.42(15)	2.59(12)	0.28
ZnO (wurtzite)	4.2(19)	2.33(28)	3.21(19)	0.015
MgO	1.33(56)	0.90(8)	2.84(15)	0.033

Supplemental References

1. Seo, H., Falk A.L., Klimov P.V., Miao K.C., Galli G., Awschalom D.D. Quantum decoherence dynamics of divacancy spins in silicon carbide. *Nat. Commun.* **7**, 1-9 (2016).
2. Maze, J.R., Dreau A., Waselowski V., Duarte H., Roch J.F., Jacques V. Free induction decay of single spins in diamond. *New. J. Phys.* **14**, 103041 (2012).
3. Zhao, N., Hu J.L., Ho S.W., Wan J.T.K., Liu R.B. Atomic-scale magnetometry of distant nuclear spin clusters via nitrogen-vacancy spin in diamond. *Nat. Nanotechnol.* **6**, 242-246 (2011).
4. Hall, L.T., Cole J.H., Hollenberg L.C.L. Analytic solutions to the central-spin problem for nitrogen-vacancy centers in diamond. *Phys. Rev. B* **90**, 075201 (2014).
5. Mims, W.B. Envelope Modulation in Spin-Echo Experiments. *Phys. Rev. B* **5**, 2409 (1972).
6. Yang, W., Liu R.B. Quantum many-body theory of qubit decoherence in a finite-size spin bath. *Phys. Rev. B* **78**, 085315 (2008).
7. Hall, L.T., Hill C.D., Cole J.H., Hollenberg L.C.L. Ultrasensitive diamond magnetometry using optimal dynamic decoupling. *Phys. Rev. B* **82**, 045208 (2010).
8. Ye, M., Seo H., Galli G. Spin coherence in two-dimensional materials. *Npj Comput. Mater.* **5**, 44 (2019).
9. Weber, J.R., Koehl W.F., Varley J.B., Janotti A., Buckley B.B., Van de Walle C.G., Awschalom D.D. Quantum computing with defects. *Proc. Natl. Acad. Sci. U.S.A.* **107**, 8513-8518 (2010).
10. Heremans, F.J., Yale C.G., Awschalom D.D. Control of Spin Defects in Wide-Bandgap Semiconductors for Quantum Technologies. *Proc. IEEE* **104**, 2009-2023 (2016).
11. Yang, L.P., Burk C., Widmann M., Lee S.Y., Wrachtrup J., Zhao N. Electron spin decoherence in silicon carbide nuclear spin bath. *Phys. Rev. B* **90**, 241203(R) (2014).
12. Mims, W.B. Phase Memory in Electron Spin Echoes Lattice Relaxation Effects in CaWo4 - Er,Ce,Mn. *Phys. Rev.* **168**, 370-389 (1968).
13. Petersen, E.S., Tyryshkin A.M., Morton J.J.L., Abe E., Tojo S., Itoh K.M., Thewalt M.L.W., Lyon S.A. Nuclear spin decoherence of neutral P-31 donors in silicon: Effect of environmental Si-29 nuclei. *Phys. Rev. B* **93**, 161202(R) (2016).
14. Balian, S.J., Wolfowicz G., Morton J.J.L., Monteiro T.S. Quantum-bath-driven decoherence of mixed spin systems. *Phys. Rev. B* **89**, 045403 (2014).
15. Maze, J.R., Taylor J.M., Lukin M.D. Electron spin decoherence of single nitrogen-vacancy defects in diamond. *Phys. Rev. B* **78**, 094303 (2008).
16. Son, N.T., Anderson C.P., Bourassa A., Miao K.C., Babin C., Widmann M., Niethammer M., Ul Hassan J., Morioka N., Ivanov I.G., Kaiser F., Wrachtrup J., Awschalom D.D. Developing silicon carbide for quantum spintronics. *Appl. Phys. Lett.* **116**, 190501 (2020).
17. Wolfowicz, G., Anderson C.P., Diler B., Poluektov O.G., Heremans F.J., Awschalom D.D. Vanadium spin qubits as telecom quantum emitters in silicon carbide. *Sci. Adv.* **6**, eaaz1192 (2020).
18. Christle, D.J., Klimov P.V., Casas C.F.D.L., Szasz K., Ivady V., Jokubavicius V., Hassan J.U., Syvajarvi M., Koehl W.F., Ohshima T., Son N.T., Janzen E., Gali A., Awschalom D.D. Isolated Spin Qubits in SiC with a High-Fidelity Infrared Spin-to-Photon Interface. *Phys. Rev. X* **7**, 021046 (2017).
19. Trifunovic, L., Pedrocchi F.L., Loss D. Long-Distance Entanglement of Spin Qubits via Ferromagnet. *Phys. Rev. X* **3**, 041023 (2013).
20. Ohno, H., Chiba D., Matsukura F., Omiya T., Abe E., Dietl T., Ohno Y., Ohtani K. Electric-field control of ferromagnetism. *Nature* **408**, 944-946 (2000).
21. Chiba, D., Fukami S., Shimamura K., Ishiwata N., Kobayashi K., Ono T. Electrical control of the ferromagnetic phase transition in cobalt at room temperature. *Nat. Mater.* **10**, 853-856 (2011).
